# *Saccharomyces cerevisiae* as host for the recombinant production of polyketides and nonribosomal peptides

**DOI:** 10.1186/s12934-021-01650-y

**Published:** 2021-08-19

**Authors:** Anna Tippelt, Markus Nett

**Affiliations:** Department of Biochemical and Chemical Engineering, Laboratory of Technical Biology, TU Dortmund University, Emil-Figge-Strasse 66, 44227 Dortmund, Germany

**Keywords:** Heterologous expression, Metabolic engineering, Natural products, Nonribosomal peptide synthetase, Polyketide synthase, *Saccharomyces cerevisiae*, Yeast

## Abstract

As a robust, fast growing and genetically tractable organism, the budding yeast *Saccharomyces cerevisiae* is one of the most widely used hosts in biotechnology. Its applications range from the manufacturing of vaccines and hormones to bulk chemicals and biofuels. In recent years, major efforts have been undertaken to expand this portfolio to include structurally complex natural products, such as polyketides and nonribosomally synthesized peptides. These compounds often have useful pharmacological properties, which make them valuable drugs for the treatment of infectious diseases, cancer, or autoimmune disorders. In nature, polyketides and nonribosomal peptides are generated by consecutive condensation reactions of short chain acyl-CoAs or amino acids, respectively, with the substrates and reaction intermediates being bound to large, multidomain enzymes. For the reconstitution of these multistep catalytic processes, the enzymatic assembly lines need to be functionally expressed and the required substrates must be supplied in reasonable quantities. Furthermore, the production hosts need to be protected from the toxicity of the biosynthetic products. In this review, we will summarize and evaluate the status quo regarding the heterologous production of polyketides and nonribosomal peptides in *S. cerevisiae*. Based on a comprehensive literature analysis, prerequisites for a successful pathway reconstitution could be deduced, as well as recurring bottlenecks in this microbial host.

**Supplementary Information:**

The online version contains supplementary material available at 10.1186/s12934-021-01650-y.

## Background

Polyketides and nonribosomal peptides encompass diverse groups of specialized molecules that are produced by bacteria, fungi, plants, and marine organisms [1, 2]. Their natural functions comprise the adaptation to environmental changes, the defense of resources or habitats, self-protection against predators and herbivores, as well as inter- and intraspecific communication [3]. Because many of these compounds possess potent antibiotic, immunosuppressive or antiinflammatory properties, they represent important resources for therapeutic drugs [4].

The development of economically feasible manufacturing processes remains an ambitious challenge for many of these compounds. Due to their complex molecular scaffolds, chemical syntheses of polyketides and nonribosomal peptides can be tortuous. The need to introduce and later remove protecting groups, the use of precious metal catalysts or toxic reagents affects production costs as well as sustainability [5, 6]. On the other hand, biotechnological approaches can be hampered by low yields and purification difficulties [7]. In many cases, the natural producers of polyketides and nonribosomal peptides show unfavorable process properties, which impede their industrial application. Examples are instable production due to long cultivation times [8] or high sensitivity against shear stress in stirred tank reactors, as observed for many filamentous and mycelia-forming microorganisms [9]. One way to overcome such issues is to reconstitute the biosynthesis of the target molecule in an industrially proven host organism. Microorganisms like *Escherichia coli*, *Saccharomyces cerevisiae*, or *Aspergillus nidulans* are frequently exploited as heterologous hosts to establish production processes for different chemical compounds [10–13].

The single-celled ascomycete *S. cerevisiae* is one of the most prominent microbial workhorses in academia and industry. As a robust, fast growing and safe organism, encoding no toxic or viral genes, budding yeast is of particular interest for biotechnological applications. The ease of transformation with exogenous DNA in conjunction with extremely efficient homologous recombination capabilities make *S. cerevisiae* a primary choice for the recombinant production of pharmaceutical drugs and other high value chemicals. Over the years, a number of techniques have been developed for the genetic engineering of *S. cerevisiae*, which exploit homologous recombination and are used for genome editing and pathway reassembly. Examples include transformation-associated recombination cloning [14–18], long terminal repeat-guided cloning [19, 20] and CRISPR/Cas9 [14, 21–23]. This arsenal has been expanded by many plug-and-play tools, which facilitate the assembly and expression of large DNA fragments [18, 20, 22, 24–32]. Other scientific breakthroughs include the development of bidirectional expression plasmids [33, 34] and synthetic minimal expression systems for *S. cerevisiae* [35–37], which make pathway refactoring in this model organism feasible.

The use of yeast as a heterologous host was pioneered in the 1980s when strains were constructed for the manufacturing of pharmaceutical proteins, like interferon-α [38, 39] and insulin [39, 40]. At this time, the exploration and comprehension of yeast’s fundamental secretory expression pathways paved the way to a successful maturation and secretion of recombinant proteins. Of particular relevance in this context is the leader sequence of the mating type peptide pheromone α-factor in *S. cerevisiae*, which was found to convey secretory competence to a multitude of heterologously expressed fusion proteins, including interferon-α and insulin [38, 40, 41]. These fusion proteins are processed by proteolytic enzymes of the secretory pathway, which remove the leader sequence prior to secretion of the mature heterologous protein [39, 42].

Not all pharmaceutically relevant polypeptides that are produced with yeast originate from humans. The expression of viral proteins, such as the Hepatitis B surface antigen [43], led to the development of the first recombinant vaccines. Nowadays, *S. cerevisiae* and the methylotrophic yeast *Pichia pastoris* are preferred hosts in vaccine development and are also used for the expression of protozoal proteins and tumor-associated antigens [44, 45].

In contrast to pharmaceutical proteins, it was not until the beginning of the twenty-first century that yeast was explored as a production platform for small molecules, including chemical feedstocks, biofuels, food additives, flavors, and cosmetics [42]. Due to its innate metabolism, yeast produces several intermediates of commercial value, such as ethanol and glycerol. The same applies, however, for many microorganisms. What makes *S. cerevisiae* particularly appealing is its long established industrial use as well as the available *omics* data for this organism, which facilitates rational metabolic engineering on the basis of mathematical models [42, 46–52]. The implementation of artificial pathways is also feasible and allows the production of molecules that do not naturally occur in yeast. An illustrative example is that of enantiopure lactic acid, which serves as raw material for the production of polylactide polymers [53]. Significant efforts were invested to establish competitive production rates in recombinant *S. cerevisiae* strains and to develop a manufacturing process at a commercial scale [54, 55].

Due to their molecular weight, polyketides and nonribosomal peptides can also be ascribed to the small molecules. However, these compounds are distinguished by highly complex chemical structures, which permit selective binding to biological targets and receptors. Interestingly, the two natural product classes feature a unifying assembly mechanism which, together with their production in yeast, will be covered in this review. Our contribution complements previous publications in the field [28, 47, 56, 57], which focus mainly on heterologous expression as a means for natural product discovery and enzyme characterization. For this reason, special emphasis will be placed on metabolic engineering aspects that are specific for polyketide and nonribosomal peptide biosynthesis. Furthermore, we provide a comprehensive and up-to-date overview of the recombinantly made polyketides and nonribosomal peptides, including the achieved titers.

## Main text

### Enzymology of polyketide and nonribosomal peptide biosynthesis

#### Polyketide synthases

In nature, polyketides are enzymatically formed by consecutive Claisen condensation reactions of short chain acyl derivatives. On the biochemical level, the assembly of polyketides is very much reminiscent of fatty acid biosynthesis, although it involves a larger variety of starter and extender units. Moreover, it shows an increased flexibility in the reductive processing of these building blocks [58]. Due to these peculiarities, polyketides exhibit a tremendous structural diversity, which ranges from polyenes, polyethers, and enediynes to macrolides, phenolic as well as polycyclic aromatic compounds.

The enzymes, which are responsible for the biosynthesis of these molecules, are called polyketide synthases (PKSs). Based upon their architecture, they can be divided into three classes [3]. Type I PKSs are large, modularly organized proteins of microbial origin. They possess multiple catalytic domains with specific functions. While most bacterial type I PKSs follow a sequential assembly logic, their fungal counterparts typically operate in a repetitive fashion. The latter is also true for type II PKSs, which form complexes of monofunctional proteins. Up to now, type II PKSs have only been found in few prokaryotic groups, *e.g.*, in actinomycete bacteria. In contrast, the type III PKSs represent the most widely distributed class of all PKSs with members known from bacteria, fungi, (micro-)algae and plants. Structurally, they are much smaller and less complex than the other two PKS classes. They consist of a homodimeric ketosynthase, which governs the entire assembly process from substrate discrimination to chain elongation and product release. In the following, we will focus exclusively on the assembly mechanisms of type I PKSs. Readers who want to learn more about type II and type III PKSs are referred to the reviews by Wang et al*.* [59] and Shimizu et al*.* [60].

**Fig. 1:**
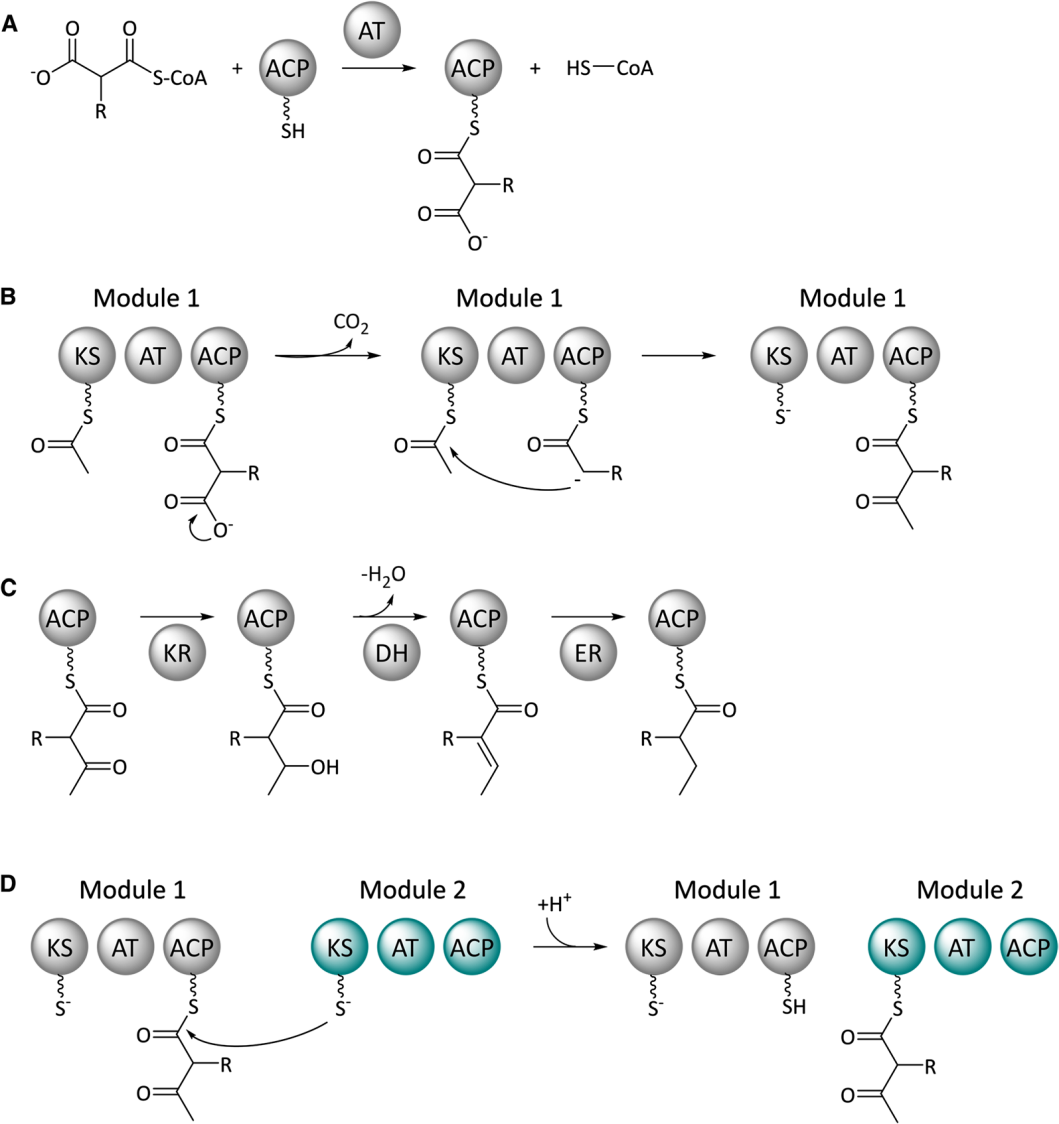
Substrate selection (**A**), chain elongation (**B**), reductive processing (**C**), and intermediate transfer (**D**) by type I PKSs. Domain notation: *KS* β-ketoacylsynthase, *AT* acyltransferase, *ACP* acyl carrier protein, *KR* ketoreductase, *DH* dehydratase, *ER* enoyl reductase

Type I PKSs can be easily distinguished by their modular architecture. Each module represents an operational unit that catalyzes the incorporation of a specific acyl building block into the growing polyketide chain and, if applicable, also the reduction of this moiety. For the elongation of a polyketide chain, every module requires three catalytic domains. The acyltransferase (AT) domain selects the correct substrate from a pool of cellular acyl-CoAs and transfers it onto the acyl carrier protein (ACP) domain (Fig. 1A). For this, the ACP domain must have undergone prior phosphopantetheinylation to provide a thiol group for the binding of the substrate (see “Enzymes for the posttranslational activation of PKSs and NRPSs” section). Afterwards, the β-ketoacylsynthase (KS) domain mediates the intrinsic Claisen condensation between the ACP-tethered substrate and the previously formed, KS-bound polyketide intermediate. This reaction is accompanied by a decarboxylation and results in the formation of a β-ketoacyl thioester (Fig. 1B).

Modules can feature up to three additional domains for the consecutive reduction of the β-keto functionality. A ketoreductase (KR) generates a β-hydroxyacyl and in conjuction with a dehydratase (DH) an α,β-unsaturated acyl intermediate. The action of an enoyl reductase (ER) eventually leads to a fully saturated product (Fig. 1C). Other than the KS, AT and ACP domains that are indispensable for the chain elongation, the reductive domains are optional. The fact that PKSs do not necessarily carry out a full reductive cycle after the addition of an extender unit like fatty acid synthases is a strong driver for the product diversity associated with these enzymes. Once all domains have performed their respective function, the ACP-bound intermediate is forwarded to the KS domain of the next module, where another chain elongation takes place (Fig. 1D). This process is concluded when a so-called termination module with a C-terminal thioesterase (TE) domain is reached. Following a final elongation step, the TE domain detaches the ACP-bound product by hydrolysis or lactonization. Afterwards the polyketide can be subject to further PKS-independent enzymatic modifications, such as glycosylations, halogenations, and alkylations.

A noteworthy deviation from the described assembly procedure is observed in fungal type I PKSs. Although these enzymes share the multidomain architecture of bacterial type I PKSs, they comprise only a single module. This module catalyzes a defined number of chain elongations. This means that its domains are used repetitively. In addition to the previously introduced domains, fungal PKSs possess characteristic domains for starter unit loading (SAT, starter unit AT domain), chain length control (PT, product template domain), and *C*-methylation (C-MeT domain). Based upon their reductive behavior, the iteratively acting fungal enzymes are grouped into non-reducing (NR), partially reducing (PR) and highly reducing (HR) PKSs. Further information on these sophisticated catalysts and their programming can be found in the reviews by Herbst et al*.* [61] and Cox [1].

#### Nonribosomal peptide synthetases

**Fig. 2:**
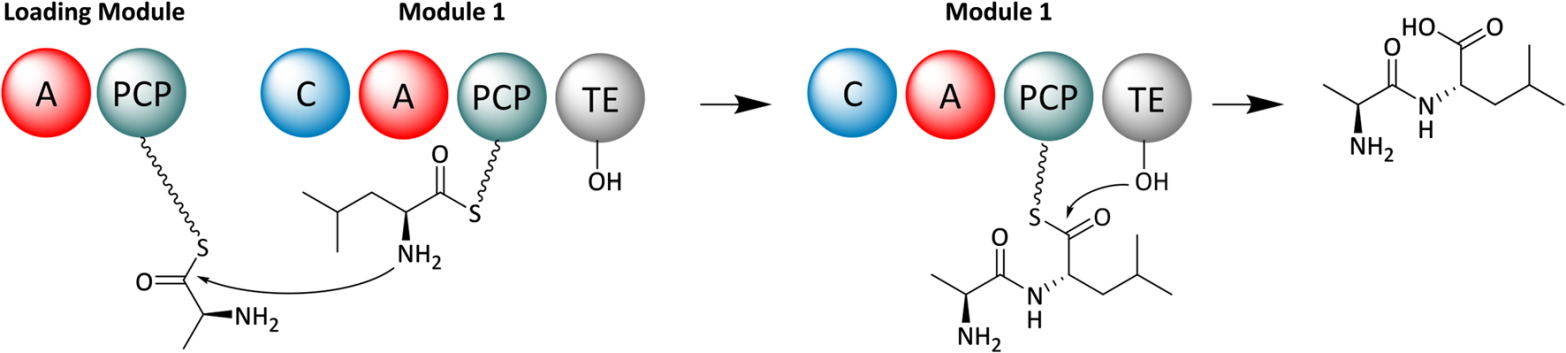
Exemplary assembly of a dipeptide by a NRPS. Domain notation: *A* adenylation, *C* condensation, *PCP* peptidyl carrier protein, *TE* thioesterase

Many bioactive peptides of microbial origin, such as the antibiotic penicillin [62] or the mycotoxin rhizonin [63], are not assembled by ribosomes. Instead the biosynthesis of these molecules is conducted by large enzymes, which are known as nonribosomal peptide synthetases (NRPSs) [64]. The mechanisms underlying NRPS biosynthesis have very much in common with the previously introduced PKSs. NRPSs are organized in modules, each of which harbors a defined set of catalytic domains [64]. Adenylation (A) domains correspond functionally to the AT domains from PKSs. They are responsible for substrate recognition and delivery. Peptidyl carrier protein (PCP) domains hold the substrate monomers during the assembly process, which is mediated by condensation (C) domains. Eventually, a TE domain terminates the biosynthesis and the product is released either as a linear peptide (Fig. 2) or as a macrolactam. A reductive release as aldehyde or alcohol is also possible, but requires a reduction (R) domain instead of a TE domain.

The main differences between PKSs and NRPSs are due to the building blocks that are utilized in the respective biosyntheses. The primary substrates of NRPSs are l-amino acids and not acyl-CoAs. Moreover, some A domains are known to be specific for aryl carboxylic acids or α-keto acids [65–67]. In any case, NRPS precursors need to be activated in an ATP-driven reaction by the A domain before they can be attached to phosphopantetheinylated PCP domains. The actual condensation leads to the formation of an amide or ester bond and not to a Claisen product. Similar to the reductive domains in PKSs, there exist also non-essential domains in NRPSs, which take part in the modification of the product structure. For example, methyltransferase (MT) domains are used for site-specific methylations, while KR domains accomplish the reduction of incorporated α-keto acids. Epimerization (E) domains, which switch the configuration of amino acid monomers from d to l, are frequently found in NRPS modules.

Of note, nature also uses NRPSs and type I PKSs in a combinatorial fashion. The unifying concept of carrier protein-based chain elongation likely contributed to the evolution of hybrid systems, which switch from PKS to NRPS interfaces and vice versa. Depending on the organization of the PKS and NRPS modules in discrete (standalone) or tethered polypeptides, these hybrid interfaces can be further classified [68]. The diversity of PKS-NRPS assembly lines is indeed remarkable and the same is true for the structures of the associated natural products, which encompass a number of medically relevant drugs [69–71].

### Prerequisites for the production of polyketides and nonribosomal peptides in *S. cerevisiae*

#### Enzymes for the posttranslational activation of PKSs and NRPSs

The functional reconstitution of PKS and NRPS pathways requires the posttranslational activation of ACP and PCP domains into their respective functional *holo*-form. This posttranslational modification step is carried out by dedicated phosphopantetheinyl transferases (PPTases). The phosphopantetheinylation reaction is Mg^2+^-dependent and involves the transfer of the phosphopantetheine moiety from coenzyme A (CoA) onto a conserved serine residue in the carrier protein. For this, the PPTases catalyze the nucleophilic side chain attack of the carrier protein serine on the 5’-pyrophosphate bond of CoA. The covalently tethered phosphopantetheine arm is now capable of binding biosynthetic intermediates through a reactive thioester bond. Furthermore, the innate flexibility of the phosphopantetheine arm facilitates the transport of bound substrates onto distal catalytic centers of the megasynth(et)ases [72, 73].

PPTases are ubiquitous to all domains of life. Although PPTases from different organisms share only low levels of sequence homology, they can be classified into two major groups. The AcpS-type PPTases are mainly involved in fatty acid biosynthesis, whereas the Sfp-type PPTases are found in secondary metabolism [65, 73, 74]. *S. cerevisiae* is no natural producer of polyketides or nonribosomal peptides and, accordingly, lacks an inherent Sfp-type PPTase for the posttranslational activation of PKSs and NRPSs. The reconstitution of a broad-spectrum PPTase is hence obligatory for the heterologous production of polyketides and nonribosomal peptides in yeast [24, 75, 76]. Sfp-type enzymes that are commonly applied for this purpose include Sfp from *Bacillus subtilis* [77], Svp from *Streptomyces verticillus* [78]*,* as well as NpgA from *Aspergillus nidulans* [79]. Still, NpgA is preferentially used for the heterologous biosynthesis of fungal natural products in yeast [80], which is likely due to its fungal origin. Although Sfp allows the reconstitution of fungal polyketide pathways [75, 81], its use is mostly reported for the activation of bacterial enzymes, as exemplified in indigoidine [82] and 1-octanol production [83].

#### Precursors for polyketide biosynthesis

Another critical factor for successful pathway reconstruction is precursor supply. Acyl-CoA units are the building blocks of PKS biosynthesis. A sufficient supply of these molecules is hence needed for the heterologous production of polyketides in yeast. Various studies reported on the engineering of acetyl-CoA metabolism to improve the availability of this metabolite in the cytosol. Proven approaches include the overexpression of the genes in the endogenous cytosolic acetyl-CoA biosynthesis pathway, which increases the metabolic flux from ethanol via acetaldehyde to cytosolic acetyl-CoA [84], or the introduction of heterologous routes for acetyl-CoA generation [85]. Other PKS substrates with a limited cellular pool include propionyl-CoA and the important PKS extender units malonyl-CoA and methylmalonyl-CoA [86, 87]. While propionyl-CoA is at least known to occur in yeast’s mitochondrial threonine catabolism [86, 88, 89], methylmalonyl-CoA is no natural metabolite of *S. cerevisiae* [86, 88–90]. Therefore, the expansion of the acyl-CoA precursor pool is vital. To address this bottleneck and achieve enhanced acyl-CoA precursor levels, three fundamental strategies can be pursued. These strategies include (i) the manipulation of endogenous acyl-CoA pathways by repressor deregulation, silencing of degradation pathways, or overexpression approaches, (ii) the feeding of acyl-CoA pathway precursors such as propionate, malonate or activated N-acetylcysteamine thioesters (SNAC-esters), and (iii) the refactoring of non-native pathways for methylmalonyl-CoA, propionyl-CoA and butyryl-CoA biosynthesis [75, 86, 90].

In the focus of malonyl-CoA engineering is the enzyme acetyl coenzyme A carboxylase ACC1, which is responsible for the biosynthesis of malonyl-CoA from acetyl-CoA in a biotin and ATP-dependent reaction. Overexpression of *acc1* by promoter exchange was reported to increase the malonyl-CoA pool in *S. cerevisiae* [87]. Furthermore, site-directed mutagenesis and subsequent deregulation of ACC1 enabled an improvement of polyketide biosynthesis [91, 92]. A different approach involves the heterologous expression of the codon optimized malonyl-CoA synthetase MatB from *Rhizobium trifolii* [28, 93]. Due to its substrate promiscuity MatB was also successfully used for the biosynthesis of methylmalonyl-CoA upon methylmalonate feeding [75]. The same precursor is also accessible through the reconstitution of a propionyl-CoA dependent methylmalonyl-CoA biosynthesis pathway. For this, the propionyl-CoA carboxylase (PCC) pathway was assembled in yeast by the introduction of three genes encoding a propionyl-CoA synthetase (PrpE), a transcarboxylase subunit (PccB) and a biotin carrier protein/biotin carboxylase subunit (AccA). This resulted in an efficient biosynthesis of methylmalonyl-CoA upon propionate feeding [75].

Following the successful reconstruction of the PCC pathway in *S. cerevisia*e the intracellular propionyl-CoA level could be further raised through a combination of promoter exchange of PrpE, propionate feeding and deletion of the (methyl)citrate synthase genes *cit2/3* to impede degradation [86]. The implementation of an artificial reverse β-oxidation pathway via yeast’s native acetoacetyl-CoA pathway and deletion of the degradative fatty acyl-CoA oxidase Pox1 gave access to butyryl-CoA, thereby expanding the diversity of precursor molecules for polyketide biosynthesis in yeast [86, 94, 95].

One of the most intensive efforts to optimize yeast for secondary metabolite biosynthesis was performed by Keasling and coworkers, who generated a recombinant yeast producing multiple short-chain acyl-CoA esters [90]. For this purpose, they introduced pathways for the biosynthesis of isovaleryl-CoA, propionyl-CoA, butyryl-CoA and hexanoyl-CoA into *S. cerevisiae*. Moreover, they optimized the production of the extender unit methylmalonyl-CoA. Isovaleryl-CoA biosynthesis was implemented by reconstitution of a bacterial 3-methylglutaconyl-CoA hydratase (LiuC), a glutaconate-CoA transferase (AibA/B) and its corresponding dehydrogenase (AibC) harnessing yeast’s native acetoacetyl-CoA pool. Butyryl- and hexanoyl-CoA biosynthesis were established from acetyl-CoA by integration of the bacterial enzymes β-ketothiolase (BktB), 3-hydroxyacyl-CoA dehydrogenase (PaaH1), crotonase (Crt), and trans-enoyl-CoA reductase (Ter). For propionyl- and methylmalonyl-CoA biosynthesis, two pathways were engineered starting from malonyl-CoA or propionate, respectively, without the necessity of an external propionate or methylmalonate feed. For this, the genes for malonyl-CoA reductase (Mcr_Ca_), 3-hydroxypropionyl-CoA synthase (3Hpcs), acryloyl-CoA reductase (Acr), 3-hydroxypropionyl-CoA dehydratase (3Hpcd), as well as the PrpE and PCC complex (PccB/AccA) had to be expressed. A propionyl-CoA independent methylmalonyl-CoA biosynthesis was established with a crotonyl-CoA carboxylase/reductase (Ccr_Ca_) from *Caulobacter crescentus* replacing the Acr-PrpE-PCC pathway [90].

The presented examples show that acyl-CoA engineering involves extensive interventions in yeast's primary metabolism. Since this rewiring affects cofactor and redox equivalents availability as well as ATP supply, additional rebalancing must be considered for an optimal pathway reconstruction [96]. The Crabtree effect, *i.e.* the redirection of the glycolytic flux towards ethanol under aerobic conditions [97, 98], deserves particular attention in this context. The Crabtree effect withdraws carbons from cytosolic acetyl-CoA biosynthesis and, thus, its elimination would be desirable for heterologous polyketide production in *S. cerevisiae*. Although ethanol formation can be impaired by targeted inactivation of corresponding alcohol dehydrogenases, this intervention also affects growth and glucose utilization due to an accumulation of acetaldehyde and acetate [85, 99]. It is important to mention that such trade-offs between growth and precursor supply are not uncommon. In Crabtree-negative yeasts, the detrimental effects on growth can be at least partially relieved by improving the acetate to acetyl-CoA conversion and the consumption of acetyl-CoA [84, 85].

#### Precursors for nonribosomal peptide biosynthesis

In nonribosomal peptide synthesis, amino acids represent the main building blocks. Accordingly, an adequate supply of amino acid units for the heterologous production of nonribosomal peptides is required. Of particular interest are shikimate-derived amino acids and aryl carboxylic acids such as 2,3-dihydroxybenzoate (DHBA), anthranilic acid or salicylic acid, since they are often limiting constituents in NRPS biosynthesis [100–102].

The shikimate pathway in *S. cerevisiae* is subject to feedback inhibition, which occurs on multiple levels and is linked to core metabolic pathways as glycolysis and the pentose phosphate pathway [100, 103]. Significant engineering efforts have been directed towards 3-deoxy-D-arabino-heptulosonate-7-phosphate (DAHP) synthase (Aro3 and Aro4) and chorismate mutase (Aro7), which are key enzymes initiating aromatic amino acid biosynthesis from phosphoenolpyruvate (PEP) and erythrose-4-phosphate (E4P). The construction of feedback-insensitive DAHP synthase (Aro4) variants in conjunction with Aro4 and Aro7 overexpression increased the flux through the aromatic amino acid pathway [104]. The pentose phosphate pathway was found to have a lower carbon flux availability compared to the PEP pathway. Nonetheless, a sufficient supply of the rate limiting substrate E4P could be achieved by rewiring the pentose phosphate pathway [105]. Some studies identified the reduced substrate affinity of DAHP synthase towards E4P in comparison to PEP as a potential bottleneck. To address this issue, the conversion of pentose to E4P was enhanced by overexpression of transketolase (Tkl), transaldolase (Tal1) and ribose-5-phosphate ketolisomerase (Rki1) [100, 105, 106]. In addition, the deletion of glucose-6-phosphate dehydrogenase (Zwf1) blocked the oxidative branch of the pentose phosphate pathway [107]. However, diversion of the carbon flux from glycolysis towards E4P continues to be a challenging endeavor for the biosynthesis of shikimate-derived secondary metabolites. As an example, Liu and colleagues demonstrated the necessity of extensive adaptations in glycolysis, pentose phosphate pathway, and shikimate metabolism in order to assemble an artificial *para*-coumaric acid pathway from tyrosine and phenylalanine [105].

### Reconstitution of type I PKSs and NRPSs in yeast

#### Reconstitution of type I PKSs

An analysis of literature databases revealed that a number of polyketides were already successfully produced in *S. cerevisiae* (Table 1). Among them are compounds of pharmacological value, such as the cholesterol lowering agent simvastatin or the anthraquinone emodin. In addition, the biosyntheses of the chemical feedstocks 6-methylsalicylic acid (6-MSA) and orsellinic acid (OSA) were reconstituted and extensively engineered in yeast. A closer inspection of the data in Table 1 shows that with one exception the heterologously synthesized compounds derive from iteratively acting PKSs of fungal or plant origin. On the one hand, this observation might be attributed to the smaller size of the respective enzymes in comparison to multimodular, bacterial PKSs. On the other hand, it might be due to the closer relatedness between the native producer and the host. In the following, we will illustrate important developments in the heterologous production of polyketides in yeast using selected compounds as examples. We will start with OSA and 6-MSA (“Orsellinic acid and 6-methylsalicylic acid” section), which are assembled by prototypical NRPKS and PRPKS, respectively [1]. Furthermore, they turned out to be useful model compounds in heterologous expression studies due to their low structural complexity and easy detection [81, 108]. Afterwards we will highlight the studies towards the reconstitution of lovastatin biosynthesis (“Lovastatin and simvastatin” section). Lovastatin serves as example for a HRPKS-derived compound and represents a molecule of commercial interest. In “Polyketides of bacterial origin” section, the heterologous expression of bacterial PKSs will be addressed.

**Table 1:** Outline of microbial polyketides that were heterologously produced in *S. cerevisiae*, including the type of reconstituted PKS, its origin and product titer

Structural Class	Compound	PKS type, protein size, domain architecture	Origin	Product titer [mg/l]	Literature
Anthracenone	TAN-1612 and derivatives	NRPKS, 1794 aa, 194.9 kDa, SAT-KS-AT-PT-ACP	Fungal	8–10	[109]
Anthraquinone	Emodin	NRPKS, 1760 aa, 191.8 kDa, SAT-KS-AT-PT-ACP	Fungal	592.5	[110]
Anthraquinone	Endocrocin	NRPKS, 1760 aa, 191.8 kDa, SAT-KS-AT-PT-ACP	Fungal	134.5	[110]
Anthraquinone	DMAC	Type III, 404 aa, 44.6 kDa, KS	Plant	Not reported	[111]
Benzoisochromane-quinone	Dihydrokalafungin	Type III, 404 aa, 44.6 kDa, KS	Plant	Not reported	[111]
Benzochromenone	(*nor*)-rubrofusarin	NRPKS, 2067 aa, 225.1 kDa, SAT-KS-AT-PT-ACP-TE	Fungal	0.2–1.1	[112]
Benzenediol lactone	Brefeldin A precursors	HRPKS, 2374 aa, 257.3 kDa, KS-AT-DH-ER-KR-ACP	Fungal	0.5–4	[113]
Benzenediol lactone	Monocillin II and pochonin D	HRPKS, 2383 aa, 260.2 kDa, KS-AT-DH-core-ER-KR-ACP NRPKS, 2090 aa, 228.6 kDa, SAT-KS-AT-PT-ACP-TE	Fungal	1.3–15	[114]
Benzenediol lactone	7',8'-dehydro-zearalenol	HRPKS, 2349 aa, 253.6 kDa, KS-AT-DH-core-ER-KR-ACP NRPKS, 2049 aa, 222.9 kDa, SAT-KS-AT-PT-ACP-TE	Fungal	20	[115, 116]
Benzenediol lactone	10,11-dehydro-curvularin	HRPKS, 2389 aa, 260.6 kDa, KS-AT-DH-MT*-KR*-ER-KR-ACP NRPKS, 2079 aa, 227.7 kDa, SAT-KS-AT-PT-ACP-TE	Fungal	11	[117]
Benzenediol lactone	*trans*-resorcylide, zearalane, lasicicol, 10,11-dehydro-curvularin, …	HRPKS, varied, KS-AT-DH-ER-KR-ACP NRPKS, varied, SAT-KS-AT-PT-ACP-TE	Fungal	8–9	[114, 115, 118–120]
Benzenediol lactone	Monocillin II, 10,11-dehydrocurvularin, lasicicol, lasilarin, radilarin, radiplodin	HRPKS, varied, KS-AT-DH-ER-KR-ACP NRPKS, varied, SAT-KS-AT-PT-ACP-TE	Fungal (combinatorial)	0.1–10	[118]
Benzo[*b*]xanthene	Bikaverin	NRPKS, 2036 aa, 221.5 kDa, SAT-KS-AT-PT-ACP-TE/CLC	Fungal	0.7–41	[121]
Coumarin	Mellein	PRPKS, 1786 aa, 193.5 kDa, KS-AT-DH-KR-ACP	Fungal	Not reported	[122]
Isocoumarin	de-*O*-methyldiaporthin	NRPKS, 2181 aa, 239.3 kDa, SAT-KS-AT-ACP-ACP-TE/CLC*	Fungal	0.8–1.7	[93]
Furo[2,3-*h*]- isochromene	Chaetoviridin A and cazaldehyde precursor	HRPKS, 2383 aa, 257.2 kDa, KS-AT-DH-MT-ER-KR-ACP NRPKS 2746 aa, 298.9 kDa, SAT-KS_AT-PT-MT-ACP-R	Fungal	0.5–1	[123–125]
Lactone	Triketide lactone	Type I, ~ 180 kDa, KS-AT-KR-ACP-TE	Bacterial (combinatorial)	0.5–1	[75]
Phenol	3-ethyl- and 3-propyl-phenol	PRPKS, 1775 aa, 190.7 kDa, KS-AT-DH-KR-ACP	Fungal	2.6–12.5	[86]
Phenolic acid	*m*-cresol	PRPKS, 1775 aa, 190.7 kDa, KS-AT-DH-KR-ACP	Fungal	589	[126]
Phenolic acid	5-methyl- orsellinic acid	NRPKS, 2590 aa, 283.7 kDa, SAT-KS-AT-PT-ACP-ACP-MT-TE	Fungal	Not reported	[127]
Phenolic acid	6-methyl- orsellinic acid	NRPKS, varied SAT-KS-AT-DH-ACP-MT-RED or SAT-KS-AT-DH-ACP-ACP-MT-EST	Fungal	0.5–1.7	[93]
Phenolic acid	6-methyl- salicylic acid	PRPKS, 1775 aa, 190.7 kDa, KS-AT-DH-KR-ACP	Fungal	200–2009	[80, 81, 87, 91, 126]
Phenolic acid	Orsellinic acid	NRPKS, 1728 aa, 190.2 kDa, KS-AT-PT-ACP-TE	Fungal	1.8	[93]
Statin	Monacolin L and J acid	HRPKS, 3038 aa, 335.0 kDa, KS-AT-DH-MT-ER*-KR-ACP-C	Fungal	20–75	[128–130]
Statin	Simvastatin	HRPKS, 3038 aa, 335.0 kDa, KS-AT-DH-MT-ER*-KR-ACP-C	Fungal	55 (in vitro)	[128]

An asterisk indicates an inactive domain. EST indicates an esterase/lipase. Additional information regarding the native producer organism, the properties of the expression strain, and the titer increase compared to the native producer are given in Additional file 1: Table S1

##### Orsellinic acid and 6-methylsalicylic acid

**Fig. 3:**
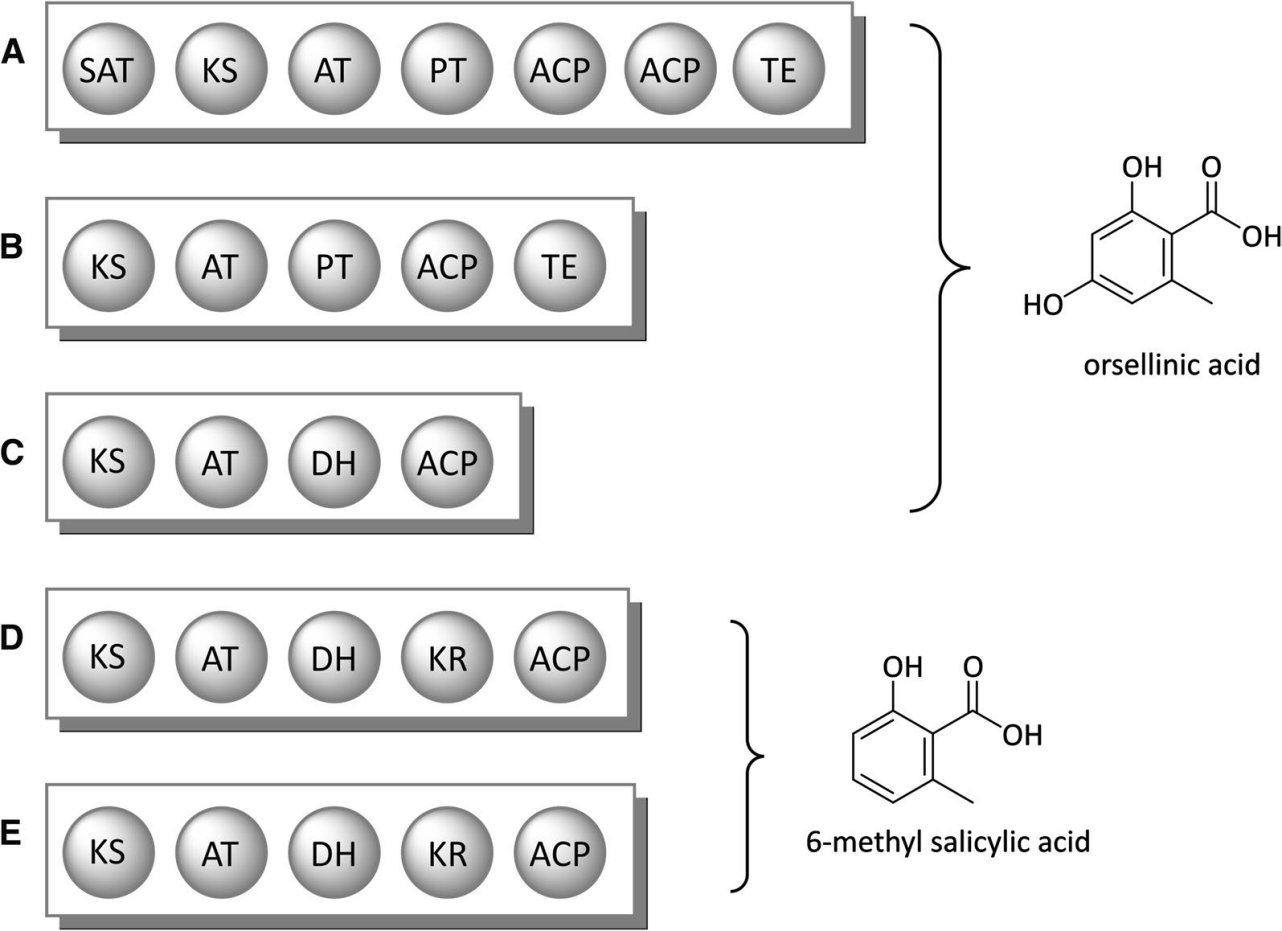
Domain architecture of OSA- and 6-MSA-forming enzymes and their products. **A** OSAS from the ascomycete *Aspergillus nidulans* [135] and the basidiomycete *Armillaria mellea* [136]; **B** OSAS from the basidiomycete *Coprinopsis cinerea* [93]; **C** OSAS from the bacterium *Micromonospora echinospora* ssp. *calichensis* [142]; **D** 6-MSAS from the ascomycete *Penicillium patulum* [143]; **E** 6-MSAS from the bacterium *Streptomyces antibioticu*s [144, 145]

Orsellinic acid (OSA) possesses potent antioxidative, neuroprotective and free radical scavenging properties [131–133] and represents a common building block in many fungal and lichen-derived secondary metabolites, such as depsides. For example, OSA is the precursor of the monoamine oxidase B inhibitor confluentic acid [134], the cathepsin K inhibitor F-9775A/B [135] and of melleolide antibiotics [136]. Its biosynthesis from one acetyl-CoA and three malonyl-CoA units is performed by the NRPKS orsellinic acid synthase (OSAS). OSAS was first isolated from *Penicillium madriti* in 1968 [137]. Since then homologs of this enzyme were identified in diverse fungal and lichen-associated ascomycetes (*e.g.*, *Aspergillus nidulans*) as well as in some basidiomycete species, among them *Coprinopsis cinerea* and *Armillaria mellea* [93, 135–138]. Structurally, OSA closely resembles 6-methylsalicylic acid (6-MSA), which is the product of a PRPKS named 6-MSA synthase (6-MSAS). However, the domain architecture of fungal OSAS is only in parts similar to 6-MSAS (Fig. 3). OSAS lacks the KR domain, but possesses two subsequent ACP-domains, which is a typical feature of fungal NRPKSs. Furthermore, the fungal OSAS exhibits an additional off-loading TE domain, which controls the chain length and the cyclization of the final product [1, 135]. The DH domain of 6-MSAS is replaced by a related PT domain, which catalyzes the regioselective aldol cyclization and aromatization of the template [135, 139–141].

Interestingly, the occurrence of OSAS and 6-MSAS is not restricted to fungi. Instead, these enzymes were also reported for several bacteria in the order Actinomycetales, where they are involved in enediyne and oligosaccharide antibiotic biosynthesis [142, 146, 147]. The domain organization in the bacterial OSAS deviates from its fungal counterpart and is actually more reminiscent of the 6-MSAS (Fig. 3) [1, 135, 144]. In fact, the bacterial OSAS only lacks the KR domain of 6-MSAS. Inactivation of the KR domain in a bacterial 6-MSAS can result in the biosynthesis of OSA instead of 6-MSA [144].

Ishiuchi et al*.* demonstrated for the first time the heterologous production of OSA and 6-methylorsellinic acid (6-MOSA) in *S. cerevisiae* with titers of 1.8 mg/l and 0.5–1.7 mg/l, respectively [93]. The achieved OSA titer with *S. cerevisiae* is lower than in the native producer *A. nidulans* (3.7 mg/l) [135], which supplies a vast pool of acyl-CoA precursor units for the biosynthesis of polyketides [148]. Therefore, a possible explanation for the lower production of OSA in yeast could be the limited availability of malonyl-CoA, which is a known bottleneck in its metabolism. Although MatB had been expressed in the heterologous host to increase its cellular malonyl-CoA level, it is evident that this approach relies on an exogenous supply of malonate [75, 129, 149]. Since the uptake of malonate is assumed to depend on passive diffusion [149–151], the concentration of available malonate in the culture medium could be a limiting factor for OSA biosynthesis in yeast. Further, the pH of the medium is of note, as it directly influences the equilibrium of the dissociated and undissociated form of malonic acid, and hence the ability of this precursor to cross the plasma membrane. A possible solution to promote malonate uptake, is the reconstitution of a functional malate permease, *e.g.*, Mae1 of *Schizosaccharomyces pombe*, which was previously demonstrated to compensate for the lack of malonate import [149, 150].

An alternative option for increasing the malonyl-CoA pool was pursued in the reconstitution of 6-MSA biosynthesis. Here, the acetyl coenzyme A carboxylase ACC1 was targeted (see “Precursors for polyketide biosynthesis” section). For instance, the Nielsen group reported a 6-MSA titer of 554 mg/l following the overexpression of ACC1 [87]. Another promising approach was described by the Da Silva group [91]. In this study, the negative regulation of ACC1 activity by the serine/threonine protein kinase Snf1 was abolished*.* As Snf1 is a globally acting regulator affecting several metabolic pathways, such as gluconeogenesis, β-oxidation and the general stress response, deletion of Snf1 was ineligible. Instead, the phosphorylation site of Snf1 in ACC1 was mutagenized, following its identification in a sequence alignment between rat and *S. cerevisiae* ACC1. In this way, ACC1 was successfully deregulated, which led to a threefold increase in 6-MSA production [91]. Noteworthy, the benefit of ACC1 deregulation with regard to malonyl-CoA supply was also independently demonstrated by the Nielsen group, who analyzed its impact on the production of fatty acid ethyl esters and 3-hydroxypropionic acid [92].

6-MSAS also served as a model enzyme to investigate the influence of bacterial (Sfp) and fungal (NpgA) PPTases on polyketide biosynthesis in *S. cerevisiae* [80]. This study revealed that the fungal PPTase NpgA outperforms Sfp in respect of 6-MSA product titers. The achieved titer was even superior to the native producers *Aspergillus terreus* and *Penicillium griseofulvum* [80]. Recently, the Boles group reported further important parameters for achieving high 6-MSA titers [126]. Initially, the group analyzed the production of this polyketide in *S. cerevisiae* using different variants of 6-MSAS, which were constitutively expressed from a 2µ multicopy plasmid. These studies revealed that the adaptation of the 6-MSAS codons to the tRNA pools in yeast has a strong positive effect on 6-MSA productivity. Furthermore, it was demonstrated that the selection of a suitable cultivation medium is crucial and should not be neglected in heterologous expression experiments. Notable product titers were only obtained in a medium supporting high cell density growth. Further experiments suggested that the production of 6-MSA is primarily limited by the availability of the corresponding PKS. This bottleneck could be relieved by combined chromosomal and episomal expression of 6-MSAS in *S. cerevisiae* [126].

The 6-MSA titers that were achieved in the aforementioned studies vary from 200 mg/l to 2 g/l and it can be assumed that this divergence is not only due to different genetic engineering strategies. Different host strains and cultivation conditions were used in these investigations, which makes a direct comparison of the reported titers difficult, if not impossible. Still, one can conclude that *S. cerevisiae* is a very promising host for the production of this polyketide, if the titers of the native producer *P. griseofulvum* and *A. terreus* (up to 0.2 mg/l) [80] or other heterologous hosts, such as *A. nidulans* (455 mg/l) [152] are taken into account. The improvements in 6-MSA biosynthesis clearly underline the great potential for the high-level production of fungal polyketides in yeast. In particular, the overexpression and deregulation of ACC1 should also be applicable to other systems, which rely on the sufficient availability of malonyl-CoA as precursor. ACC1 is the rate-limiting enzyme for the intracellular conversion of acetyl-CoA to malonyl-CoA and represents the major source of this relevant precursor for PKS biosynthesis. Other overarching concepts are codon optimization and overexpression of PKS genes. In addition, fungal PPTases are likely advantageous for the heterologous expression of fungal PKSs.

##### Lovastatin and simvastatin

Lovastatin is known as a potent cholesterol-lowering agent. Industrially, this polyketide is produced by solid state and submerged fermentation of the native producer *A. terreus*, which is a filamentous fungus and opportunistic pathogen [153]. However, alternative non-pathogenic host systems with more favorable process properties, such as *A. oryzae*, *P. pastoris* and *S. cerevisiae*, have been explored for the production of lovastatin [154].

**Fig. 4:**
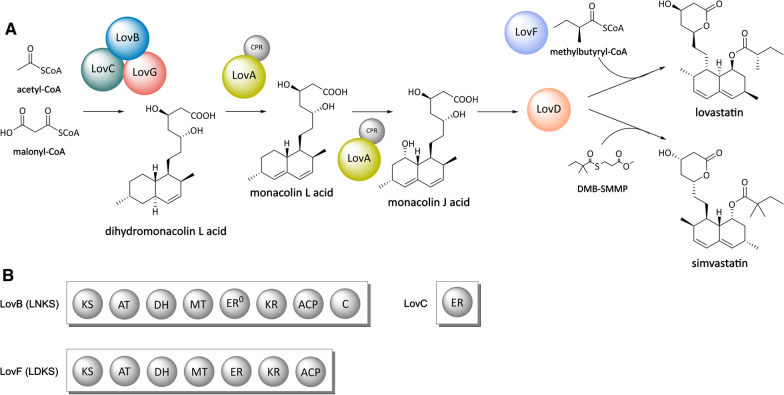
**A** Biosynthesis of lovastation and generation of its semisynthetic analogue simvastatin. *LovB* lovastatin nonaketide synthase (LNKS), *LovC* enoylreductase, *LovG* multifunctional esterase, *LovA* cytochrome P450 monooxygenase, *CPR* cytochrome P450 reductase, *LovF* lovastatin diketide synthase (LDKS), *LovD* thioesterase-like acyltransferase, *DMB-SMMP* dimethylbutyryl-S-methyl mercaptopropionate. **B** Domain architectures of LovB, LovC and LovF. *ER*^0^ dysfunctional enoylreductase

A pair of iteratively acting HRPKSs, namely the lovastatin nonaketide synthase LovB and the lovastatin diketide synthase LovF execute the biosynthesis of the two polyketide building blocks from which lovastatin is assembled. In detail, LovB synthesizes the intermediate dihydromonacolin L acid (DMLA) from malonyl-CoA and acetyl-CoA in cooperation with the enoylreductase LovC and the multifunctional esterase LovG. The DMLA intermediate is further processed by the cytochrome P450 monooxygenase LovA to form the intermediates monacolin L acid (MLA) and monacolin J acid (MJA) by successive dehydration and hydroxylation. This process is assisted by the cytochrome P450 oxidoreductase CPR, which is functioning as an electron donor enzyme to regenerate the hem-containing LovA using the reducing equivalent NADPH. Subsequently, the LovF-derived diketide methylbutyryl-CoA is linked to MJA by the action of the thioesterase-like acyltransferase LovD to yield lovastatin (Fig. 4) [129, 130, 154–156].

In an initial reconstitution attempt, the lovastatin nonaketide synthase LovB and the associated enoylreductase LovC were expressed from a plasmid in an NpgA-carrying yeast strain [157]. Although phosphopantetheinylation of LovB took place, the production of a monacolin precursor could not be observed. The absence of the corresponding metabolite was assumed to be caused by domain inactivity or impeded product shuttling. Subsequent in vitro and in vivo studies confirmed the incapability of LovB to release correctly processed compounds and demonstrated the necessity of an interacting TE domain to direct the off-loading [129, 130]. Although, TE domains from other PKSs, *e.g.*, from the enzymes engaged in hypothemycin [116] and zearalenone biosynthesis [158] were successfully used to complement the production of DMLA, the achieved product titers remained extremely low with only 400 µg/l. Further research efforts identified LovG and not LovD as the native LovB-interacting TE domain. LovG proved also to be crucial for the correct iterative function of the entire enzyme complex. Not only does this enzyme catalyze the product release, but it is also involved in the clearing of incorrectly processed intermediates from LovB, thus having an important proofreading function during chain elongation. In the contrary, the second TE-like enzyme LovD was identified to exclusively interact with the lovastatin diketide synthase LovF. The importance of these findings could be demonstrated in another expression study. Coexpression of *lovG, lovC* and *lovB* yielded a titer of 35 mg/l DMLA in vivo. Episomal coexpression of *lovB*, *lovC*, *lovG*, *lovA* and the *cpr* from *A. terreus*, which served to bypass redox limitations, eventually led to the production of the lovastatin precursor MJA with a titer of 20 mg/l [130].

Up to now, the total biosynthesis of lovastatin has not been established in yeast. However, the Tang group recently described the biotechnological production of simvastatin (Fig. 4) [128], which is a semisynthetic derivative of lovastatin. For this, the essential pathway genes for MJA biosynthesis were expressed together with *lovD* in *S. cerevisiae*. Methylbutyryl-CoA, which is required for lovastatin biosynthesis, but cannot be generated in yeast in the absence of LovF was replaced with the artificial acyl donor α-dimethylbutyryl-S-methyl-mercaptopropionate (DMB-SMMP). The latter was fed to the expression culture and, subsequently, introduced into the biosynthesis exploiting the natural substrate promiscuity of LovD. Initially, only 0.5 mg/l simvastatin were produced, but extensive engineering efforts helped to increase the polyketide yield considerably. Specifically, the authors knocked out the pyruvate carboxylase Pyc2 and the lysophospholipase Nte1, which is involved in lipid biosynthesis regulation. Furthermore, overexpression of LovA increased the conversion of DMLA to MJA, resulting in a maximum titer of 75 mg/l MJA. Another engineering target was the LovD-catalyzed reaction, which had been identified as a major bottleneck in simvastatin production. Experimental evidence indicated that pH-dependent transport limitations and toxicity issues of DMP-SMMP impeded higher conversion rates. Although adjustment of the culture pH had a positive effect on simvastatin production, the achieved titer (5.9 mg/l) was still low, corresponding to a MJA conversion of less than 15%. When DMP-SMMP was added to a freshly prepared lysate of the MJA-producing and LovD-expressing *S. cerevisiae* strain, the simvastatin titer increased up to 55 mg/l [128].

Important lessons were learnt from the reconstitution of lovastatin biosynthesis genes in yeast, which can be regarded as a showcase for complex HRPKS systems. First, TE-mediated proofreading and product release were identified as crucial factors in the overall production performance. The example of LovG shows that, contrary to PPTases, TE domains cannot be easily replaced by nonnative enzymes if high yields are to be obtained, especially when the TE has further proofreading functionalities. Secondly, PKS performance might depend on interactions with tailoring enzymes. The balancing of biocatalytic activities can pose a formidable challenge for the genetic engineer. Last, yet importantly, we note that substrate and/or product toxicity as well as transport limitations can have a considerable impact on the productivity of a heterologous host. Although such issues are not always foreseeable, they can be solved by procedural improvement, as nicely illustrated in the production of simvastatin with a yeast cell extract.

##### Polyketides of bacterial origin

The only example in which a modular type I PKS of bacterial origin was reconstituted in yeast has been reported by the Kealey group and involved the production of triketide lactone (TKL) [75]. For this, the researchers used an artificial enzyme comprising module 2 of the deoxyerythronolide B megasynthase (DEBS) fused directly to the DEBS-TE domain [159, 160]. The corresponding PKS gene was coexpressed with tRNA genes to support the sufficient translation of codons that are rarely used by yeast. In addition, refactoring of the PrpE-PCC pathway (see “Precursors for polyketide biosynthesis” section) and feeding of propionate and N-acetylcysteamine-propyl-diketide thioesters were necessary to achieve TKL production, albeit at very low titers (0.5–1 mg/l) [75].

Recently, Jakočiūnas and colleagues attempted to express the bacterial type II PKS, which is involved in actinorhodin biosynthesis, in *S. cerevisiae* [111]. Despite using codon optimized genes, no evidence for the production of actinorhodin or any of its biosynthetic intermediates was obtained. Although the actinorhodin PKS is structurally different from type I PKS systems, this finding underlines the difficulty to reconstitute bacterial PKSs in yeast. It is particularly noteworthy that Jakočiūnas and colleagues still achieved the production of a late stage actinorhodin intermediate named dihydrokalafungin in yeast by replacing the actinorhodin PKS with a plant-derived type III PKS. The latter had been described to generate a polyketide of identical chain-length as the bacterial PKS and was now successfully combined with other heterologous enzymes from the actinorhodin pathway [111].

Apart from the two aforementioned studies, we did not find further references on the production of bacterial polyketides with *S. cerevisiae*. Due to the lack of literature reports, it remains elusive if yeast actually represents a suitable host for multimodular, bacterial PKSs. In fact, the requirements to express bacterial type I PKSs in *S. cerevisiae* are multifold and still underexplored in many aspects. Dependency on external substrate supply in case of rare acyl-CoAs, expression of G + C rich genes, codon usage, coexpression of bacterial tRNAs to compensate translation bottlenecks and provision of stereoisomers for selective PKS domains are only some of the issues to consider, when switching from a bacterial to a yeast host system. Since the functional expression of bacterial PKSs in yeast seems to require extensive engineering efforts, alternative approaches, such as the use of analogous enzymes [111]*,* might be more worthwhile.

#### Reconstitution of NRPS

Similar to the situation with PKSs, *S. cerevisae* has been preferentially used as a host for NRPSs of fungal origin (Table 2). A closer analysis of the successfully reconstituted assembly lines reveals that they are distinguished by a comparatively modest size. The largest NRPSs, which were transferred to yeast, are the ACV synthetase from penicillin biosynthesis and the fumiquinazoline F-forming synthetase TqaA, with a size of 426 and 438 kDa, respectively. Although the two enzymes could be functionally expressed, the observed product titers were extremely low. Much more promising results were obtained after the reconstitution of smaller NRPS systems, featuring only one or two adenylation domains. Among them, those enzymes that deviate from the linear assembly mechanism of NRPSs were found to support particularly high product titers in yeast. Examples are the beauvericin NRPS and the atromentin synthetase. Overall, this suggests a correlation between the size of the heterologously expressed enzyme and the achievable product titer. The following paragraphs highlight the reconstitution of exemplary NRPSs. The penicillin NRPS ("Penicillin" section) was chosen to represent the enzymes following a sequential or linear assembly strategy, in which every module is recruited only once during the biosynthesis. This selection was made because of the medicinal importance of penicillin. The biosyntheses of beauvericin and of the related bassianolide are carried out by iteratively acting NRPSs. Unlike the atromentin synthetase, these enzymes feature a canonical domain architecture including C domains, which is why they are discussed in detail here (“Beauvericin and bassianolide” section). In “Indigoidine” and “Peptides derived from multimodular bacterial NRPSs” sections, we will introduce the only bacterial NRPSs that were successfully reconstituted in yeast, namely the indigoidine NRPS and a combinatorial enzyme.

**Table 2:** Outline of NRPS-derived secondary metabolites that were heterologously produced in *S. cerevisiae*, including characteristics of the reconstituted NRPS, its origin and product titer

Structural Class	Compound	NRPS assembly mode, protein size, and domain architecture	Origin	Product titer [mg/l]	References
β-Lactam	Benzylpenicillin	linear, 3791 aa, 426.0 kDa, A-PCP-C-A-PCP-C-A-PCP-E-TE	Fungal	14.9 × 10^–6^	[24, 48]
Benzodiazepine	Asperlicin C/D	iterative, 2442 aa, ~ 276 kDa, A-PCP-C-A-PCP-C	Fungal	Not reported	[161]
Benzodiazepine	Benzo-diazepinedione	linear, 2359 aa, 261.4 kDa, C-A-PCP-C-A-PCP-E	Fungal	2	[76]
Benzoquinone	Atromentin	iterative, 921 aa, 101.9 kDa, A-PCP-TE	Fungal	Not reported	[162]
Cyclic dipeptide	Tryprostatin A/B	linear, 2211 aa, 242.8 kDa, A-PCP-C-A-PCP-C	Fungal	0.1–36	[163, 164]
Cyclodepsipeptide	Bassianolide	iterative, 3147 aa, 348.3 kDa, C-A-PCP-C-A-MT-PCP-PCP-C	Fungal	21.7–26.7	[165, 166]
Cyclodepsipeptide	Beauvericin	iterative, 3190 aa, 351.9 kDa, C-A-PCP-C-A-MT-PCP-PCP-C	Fungal	33.8–105.9	[165, 166]
Dioxolane	Phenguignardic acid	iterative, 947 aa, 104.5 kDa, A-PCP-TE	Fungal	15	[162]
Dipeptide	D-Phe–L-Leu	linear, 1088 aa, 122.7 kDa, A-PCP-E linear, 1276 aa, 143.9 kDa, C-A-PCP-TE	Bacterial (combinatorial)	Not reported	[167]
Furanone	Aspulvinone E	iterative, 926 aa, 102.4 kDa, A-PCP-TE	Fungal	13	[162]
Furanone	Butyrolactone IIa	iterative, 931 aa, 102.6 kDa, A-PCP-TE	Fungal	35	[162]
Phenolic aldehyde	2,4-dihydroxy-5,6-dimethyl-benzaldehyde	iterative, 2590 aa, 283.7 kDa, SAT-KS-AT-PT-ACP-ACP-MT-TE linear, 1069 aa, 118.9 kDa, A-ACP-R	Fungal	Not reported	[127]
Pyridone	Indigoidine	iterative, 1283 aa, 141.2 kDa, A-Ox-PCP-TE	Bacterial	980	[82]
Pyridone	Preaspyridone	iterative, 3930 aa, 431.3 kDa, KS-AT-DH-MT-ER*-KR-ACP-C-A-PCP-R	Fungal	4	[168]
Quinazoline	(7-hydroxy)-fumiquinazoline F	linear, 3955 aa, 437.9 kDa, C-A-PCP-C-A-PCP-E-C-A-PCP-C	Fungal	0.4–2	[76, 93]

An asterisk indicates an inactive domain. Additional information regarding the native producer organism, the properties of the expression strain, and the titer increase compared to the native producer are given in Additional file 1: Table S2

##### Penicillin

**Fig. 5:**
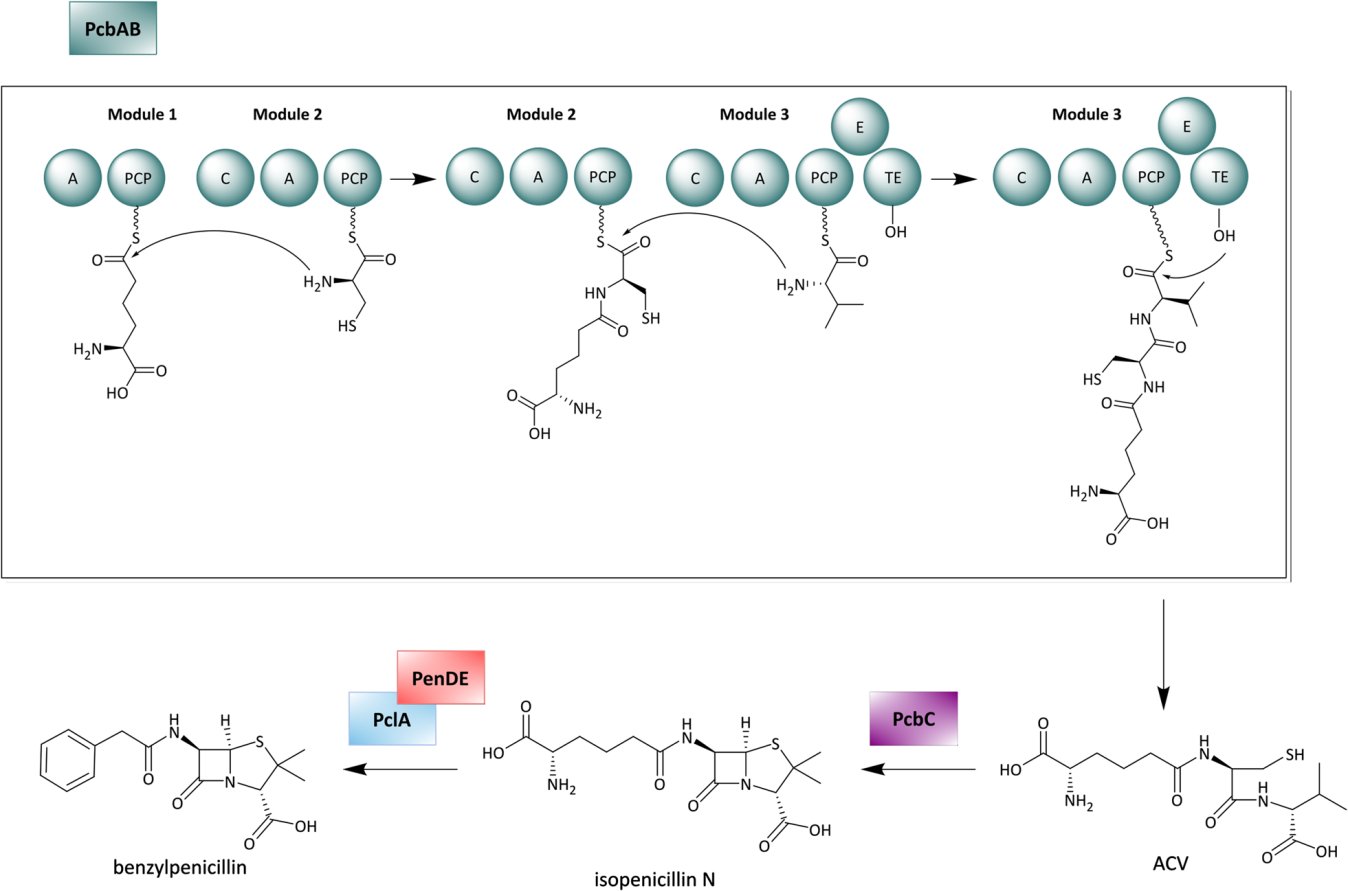
Penicillin biosynthesis in *Penicillium chrysogenum*. Domain notation: *A* adenylation, *PCP* peptidyl carrier protein, *C* condensation, *E* epimerase, *TE* thioesterase

The β-lactam benzylpenicillin (*syn.* penicillin G) is naturally produced by the filamentous fungi *Penicillium chrysogenum* and *P. notatum*. It is a potent inhibitor of bacterial cell wall biosynthesis and one of the groundbreaking discoveries of the twentieth century to treat infectious diseases. In *P. chrysogenum* the biosynthesis of penicillin involves four enzymes: PcbAB, PcbC, PclA, and PenDE. The trimodular NRPS PcbAB, which is also known as ACV synthetase, initiates the biosynthesis with the assembly of the intermediate ACV from l-α-aminoadipic acid, l-cysteine and l-valine. Subsequently, the isopenicillin N synthase PcbC catalyzes the characteristic β-lactam ring formation, which converts ACV into isopenicillin N. The final biosynthetic step is performed by the acyl-CoA-isopenicillin N acyltransferase PenDE in conjunction with the phenylacetyl CoA-ligase PclA. These two enzymes, which are located in the peroxisome of *P. chrysogenum*, are responsible for the replacement of the α-aminoadipyl moiety with a phenylacetic acid unit, converting isopenicillin N to benzylpenicillin (Fig. 5) [169].

Early attempts to refactor β-lactam biosynthesis in yeast were described by Siewers et al. [170]. The researchers used a plasmid for the combined expression of PcbAB and the PPTase NpgA, applying a galactose-inducible, bi-directional promoter system. This approach yielded low amounts of the ACV intermediate (1 µg/g dry cell weight). While initial efforts to improve this titer, including the use of different PPTases and codon optimization of PcbAB, were met with limited success, the reduction of the cultivation temperature from 30 to 20 °C increased the ACV production titer to 1 mg/g dry cell weight. This result was attributed to an improved solubility of the expressed proteins. Surprisingly, the chromosomal integration of the biosynthetic genes dramatically decreased the product yield, which was referred to a lower dosage of the biosynthesis genes in comparison to a plasmid-based expression [170].

Despite this observation, Awan et al*.* chose to integrate the *pcbAB-npgA* expression cassette into the TRP1 locus of *S. cerevisiae*, when they attempted to reconstruct the entire penicillin pathway [48]. The missing three pathway genes, *pcbC*, *pclA* and *penDE*, were expressed from a plasmid. Subsequent investigations revealed that the production of benzylpenicillin depends on correct protein sorting in the heterologous host. In the native penicillin producer *P. chrysogenum*, the two enzymes PclA and PenDE are located in the peroxisome*,* which is essential for their functionality [169, 171, 172]. The organelle is not only providing a microenvironment close to the ideal pH of these enzymes, but also enables an efficient biosynthesis by the accumulation of enzymes and substrates. A similarly high substrate concentration is not possible in the cytoplasm due to toxicity issues [169, 173].

To probe the translocation of PclA and PenDE into the yeast’s peroxisome, Awan et al*.* used fluorescence tagging and microscopy. This analysis showed that the native peroxisome targeting sequence of these two enzymes is not compatible with the protein sorting system of *S. cerevisiae*. To solve this problem, the inherent transit peptide sequences of PclA and PenDE were replaced with corresponding signal sequences of *S. cerevisiae*. This approach successfully directed both enzymes into the host’s peroxisome and led to an initial penicillin G titer of 90 pg/ml. After the expression conditions for each biosynthesis gene had been optimized by screening different promoter combinations, benzylpenicillin production reached 5 ng/ml [48]. In another study, the same research group replaced the originally applied YRp system with a YEp expression vector in an engineered yeast strain. The elevated copy number of the 2μ-derived plasmid promoted the expression of the biosynthesis genes and raised the production titer of benzylpenicillin to 14.9 ng/ml [24].

It is evident that the constructed recombinant *S. cerevisiae* strains cannot compete with industrial *P. chrysogenum* strains, which achieve titers of 40–50 g/l [174]. Nonetheless, the heterologous production of benzylpenicillin is a noteworthy achievement. Not only does it illustrate the possibility to functionally express multimodular NRPS systems in yeast, but it also highlights the importance to consider subcellular localization of biosynthesis enzymes in heterologous hosts. Furthermore, it was demonstrated once again that the balanced expression of pathway genes, the expression background and the choice of cultivation conditions each have a significant impact on the productivity of the host.

##### Beauvericin and bassianolide

**Fig. 6:**
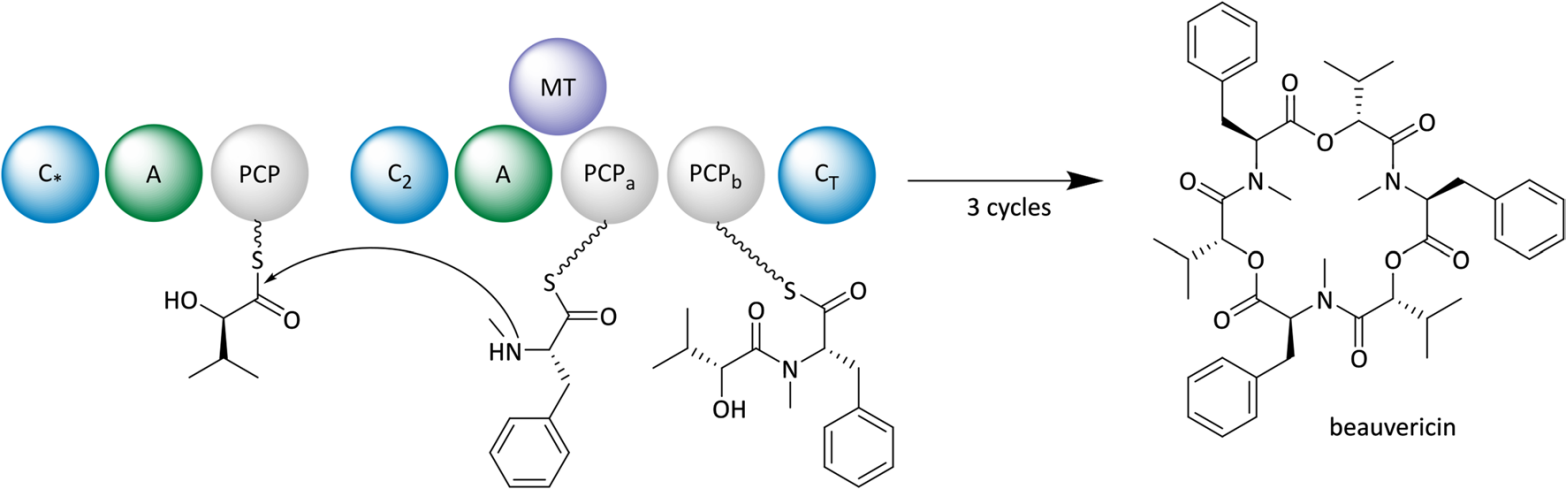
Proposed model for beauvericin biosynthesis. Domain notation: *C* condensation, *A* adenylation, *PCP* peptidyl carrier protein, *MT* *N*-methyltransferase, C* starter condensation domain, *C*_*T*_ C-terminal condensation domain

In nature, the two cyclodepsipeptides beauvericin and bassianolide are produced by the ascomycete *Beauveria bassiana* ATCC 7159. They are composed of alternating d-hydroxyisovaleric acid (d-Hiv) and *N*-methyl-amino acid units (l-phenylalanine in beauvericin or l-leucine in bassianolide biosynthesis). The NRPSs, which are involved in the cyclooligomerization of these building blocks exhibit an unusual domain architecture featuring two successive PCP domains and a C-terminal C domain (Fig. 6). This organization is likely important for their specific mode of operation. During each iterative cycle, modules 1 and 2 recruit and connect the respective monomers, though it is still unclear whether the actual oligomerization involves an elongation in dipeptidol units (parallel mode) or the successive addition of monomers (linear or looping mode). In any case, the C-terminal C domain stops the biosynthesis after a defined chain length has been reached through macrocyclization [166, 175].

From a medical perspective, beauvericin and bassianolide are interesting due to their potent antiproliferative, antifungal, antibiotic, anthelminthic and insecticidal activities. Of further note are their effects on human cancer cells by activating apoptotic pathways and inhibiting cell motility as well as metastasis [165, 176, 177]. It is therefore not surprising that these cyclodepsipeptides are attractive targets for heterologous production. Both, the beauvericin and the bassianolide NRPS, were individually expressed from a plasmid in a *S. cerevisiae* strain with a chromosomal copy of the NpgA-PPTase gene [165]. This resulted in production titers equivalent to those observed in *B. bassiana*, *i.e.*, 33.8 mg/l beauvericins and 21.7 mg/l bassianolide, respectively. Feeding of the natural precursors d-Hiv, l-phenylalanine and l-leucine improved the titer to 42.2 mg/l beauvericins and 26.7 mg/l bassianolide. To avoid expensive precursor feeding and support in situ d-Hiv formation, a pathway-associated ketoisovalerate﻿ reductase (KIVR) from *B. bassiana*, which converts l-valine into d-Hiv, was overexpressed in the beauvericin-producing *S. cerevisiae* strain. This resulted in a further product titer increase up to 105.8 mg/l [165]. Interestingly, the feeding of more than 10 mM l-valine had a detrimental effect on beauvericin biosynthesis. Overall, optimization of precursor supply and reconstitution of the pathway associated enzyme KIVR allowed the heterologous production of beauvericins and bassianolide in better titers than those observed in the native producer.

##### Indigoidine

**Fig. 7:**
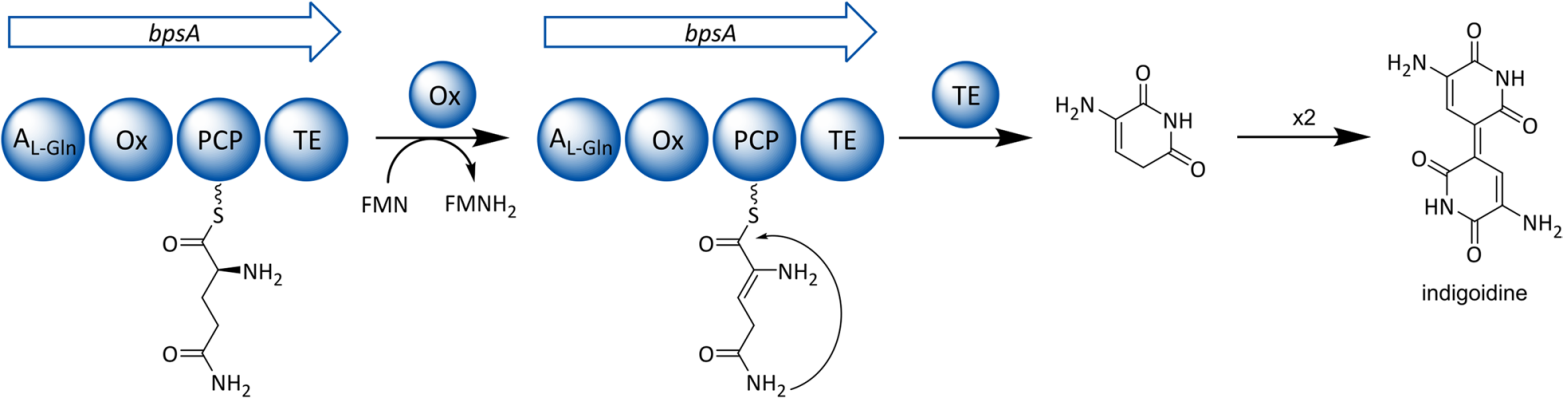
Proposed model for indigoidine biosynthesis. Domain notation: *A*_*L-Gln*_ adenylation domain selective for L-glutamine, *Ox* oxidation domain, *PCP* peptidyl carrier protein, *TE* thioesterase

A bacterial NRPS, which was functionally expressed in *S. cerevisiae*, catalyzes the production of the pigment indigoidine. The domain architecture of the indigoidine synthetase (BpsA) is even more unusual than the aforementioned beauvericin NRPS. Unlike the latter, BpsA completely lacks a C domain and consists only of an A domain with an integrated oxidation (Ox) domain, a PCP and a TE domain. According to the actual biosynthetic model, indigoidine is assembled from two l-glutamine monomers, which are individually cyclized by BpsA. Upon their TE-mediated release, the two amino acid moieties undergo a spontaneous oxidative dimerization (Fig. 7), similar to the biosynthesis of indigo from two indoxyl molecules [178]. The Ox domain in BpsA was proposed to dehydrogenate both l-glutamine monomers at the C2-C3 positions, although it is still elusive, if the oxidation occurs on PCP-bound substrates.

The blue pigment indigoidine has attracted some interest as an environmentally friendly dye, which is why its heterologous production was probed in *S. cerevisiae* [82, 179]. Briefly, a codon-optimized *bpsA* gene was stably integrated into the yeast genome using a CRISPR-Cas9 approach [82]. Furthermore, the Sfp-PPTase was integrated into the yeast’s δ-sites [180], following a multicopy-integration protocol, which increases the copy number and stabilizes the insert [181]. Although not stated in the corresponding publication, this likely improved the phosphopantetheinylation of the introduced NRPS. In sum, these efforts culminated in a yeast strain producing indigoidine.

An interesting observation was made during these investigations. Although the *bpsA* gene was constitutively expressed, pigment production was clearly delayed in comparison to biomass formation when glucose was used as a carbon source. Investigations on carbon source dependency and production dynamics revealed that indigoidine formation is linked to carbon depletion and respiration of non-fermentable carbon sources. This can be explained by an increased flux of the TCA cycle during respiration and therefore an elevated concentration of α‑ketoglutarate, which is the direct precursor of l-glutamine. Subsequently, carbon depletion was implemented into large-scale process development, using a signal-based pulse feeding strategy. After total consumption of glucose and fermentative by-products, the pulse-feed was applied to maintain the metabolic state of respiration without the loss of biomass formation. Because of discrete production timing, the indigoidine titer could be raised up to 980 mg/l. In this regard, the recombinant yeast is outperforming the native producer *Streptomyces lavendulae*, which synthesizes only 5.5 mg/l of pigment [178]. However, it should be mentioned that indigoidine has also been heterologously produced in bacterial hosts, such as *Streptomyces lividans*, *E. coli* and *Pseudomonas putida*. Especially the industrially relevant *P. putida* turned out to achieve very high product titers of up to 25.6 g/l [182].

Nonetheless, *S. cerevisiae* can be considered as a suitable host for industrial indigoidine production, although further optimization is necessary to compete with *P. putida*. The example of indigoidine illustrates that pathway reconstitution requires a profound knowledge of the metabolic state and catabolic process regulations of the host at set fermentation conditions.

##### Peptides derived from multimodular bacterial NRPSs

**Fig. 8:**
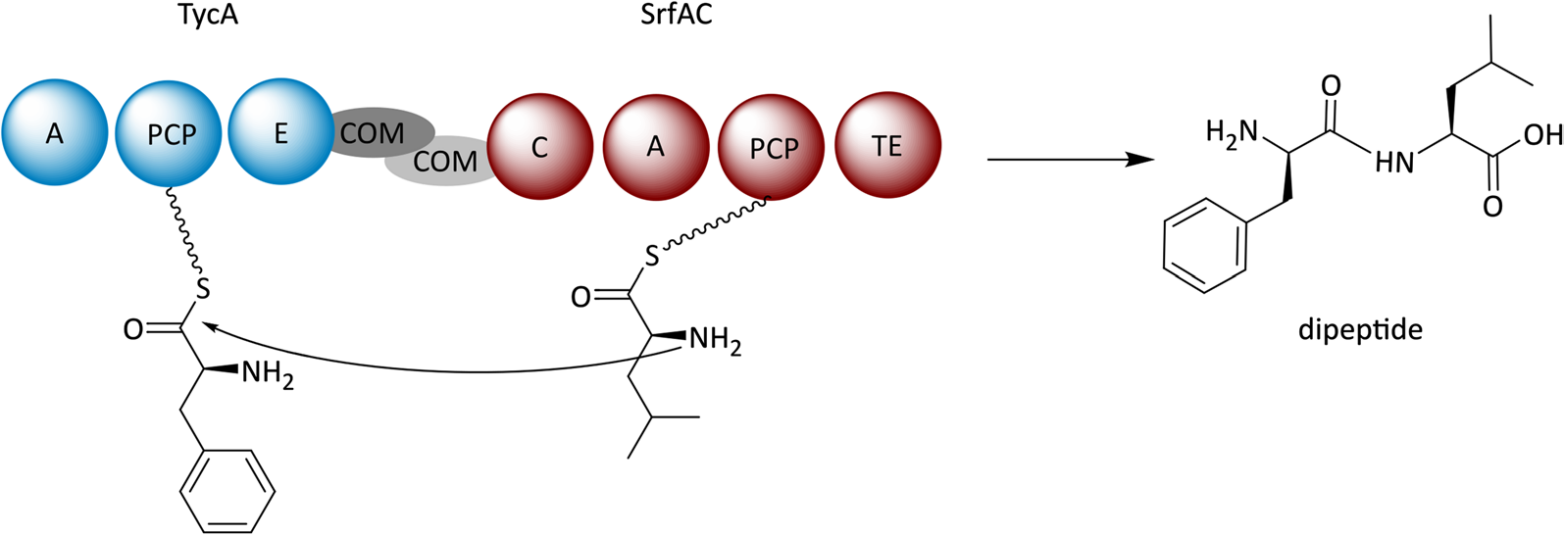
Combinatorial biosynthesis of a dipeptide using the tyrocidine (TycA) and surfactin (SrfAC) synthetases. Domain notation: *A* adenylation, *PCP* peptidyl carrier protein, *E* epimerization, *COM* communication-mediating, *TE* thioesterase

It is quite obvious that the indigoidine synthetase cannot serve as a paradigm for the expression of bacterial NRPSs in *S. cerevisiae* due to its peculiarities. In fact, information on the reconstitution of bacterial, multimodular NRPSs is scarce. A noteworthy exception is a study by Siewers et al*.*, in which modules from two different bacterial NRPSs, namely the tyrocidine synthetase (TycA) and the surfactin synthetase (SrfAC), were introduced into yeast and individually expressed from 2µ multicopy plasmids [167]. To enable a functional interaction between the TycA and SrfAC modules, they had been furnished with compatible communication-mediating (COM) domains. These domains are known to mediate the necessary protein–protein interactions in multimodular megasynthetase complexes to synergistically direct the assembly of a final product [183, 184]. The successful expression of both, TycA and SrfAC, was demonstrated by fluorescence microscopy after fusion of the NRPSs to fluorescent proteins. Furthermore, the in vivo assembly of an artificial dipeptide was shown by LC/MS analyses, which confirmed the functionality of this artificial NRPS assembly line (Fig. 8). This result is noteworthy for two reasons. First, it illustrated a useful option for combinatorial reprogramming of NRPSs. Secondly, the module splitting approach bears considerable potential for the successful reconstitution of large multimodular NRPSs in *S. cerevisiae*.

## Conclusions

The polyketides and nonribosomal peptides that were heterologously produced in *S. cerevisiae* range from small aromatic molecules like 6-MSA and orsellinic acid to highly complex molecular scaffolds (Tables 1 and 2). The structural complexity of a product has apparently no effect on the success of a reconstitution approach. Strikingly, the majority of these compounds derive from iteratively acting NRPS or PKS systems. In contrast, there are only few compounds, such as fumiquinazoline F and benzodiazepinedione, which are biosynthesized on multimodular assembly lines in a linear fashion. One reasonable explanation for this circumstance could be the size of the reconstituted megaenzymes. The iterative enzymes only comprise a minimal set of catalytic domains that are used repetitively. Due to their small size, the ribosomal synthesis of these proteins imposes a bearable metabolic burden for the cell. In the contrary, the expression of large multimodular assembly lines might interfere with the endogenous protein biosynthesis of the host. Similar assumptions have been made by Süssmuth and Mainz, who speculated that multimodular NRPSs are energetically more costly and more prone to misfolding as well as proteolysis than iterative synthetases [2]. The metabolic burden hitherto has not received much attention in pathway reconstitution approaches. However, our literature survey indicates that successful heterologous production in yeast is likely connected to the size and maybe the assembly mode (linear or iterative) of the expressed enzyme. Therefore, the extent of the metabolic burden caused by heterologous expression should be considered as a limiting factor in both, PKS and NRPS, reconstitution. Adjustments, such as tRNA pool optimization and expression temperature reduction, can promote a successful reconstitution, as exemplified in TKL [75] and ACV [170] biosynthesis. An interesting approach for multimodular pathway reconstruction might further be found in the “split-module” approach, which was tested successfully to assemble an artificial dipeptide [167].

Apart from metabolic burden constraints, our literature analysis exposed four central issues and solution concepts, which are particularly relevant for producing polyketides and nonribosomal peptides in yeast. They can be summarized as (i) sufficient availability of biosynthetic precursors, (ii) adequate phosphopantetheinylation of the PKSs and/or NRPSs, (iii) balanced expression of tailoring enzymes, and (iv) efficient expression of PKSs and NRPSs.

(i) The biosynthesis of polyketides and nonribosomal peptides undoubtedly requires a high and reliable level of precursor molecules. This can be achieved by in situ biosynthesis exploiting the primary metabolism of the host or by exogenous supply. A rate-limiting bottleneck for the biosynthesis of malonyl-CoA is the acetyl coenzyme A carboxylase ACC1. On the one hand, ACC1 represents the only intrinsic source of this relevant PKS precursor in *S. cerevisiae*. On the other hand, it is affected by a strict catabolite-directed regulation through Snf1. Promising concepts to circumvent the bottleneck of insufficient malonyl-CoA production encompass ACC1 overexpression [87] and Snf1-deregulation [91, 92]. Also, a combination of both principles can be advantageous to increase the overall production performance, as exemplified in 6-MSA biosynthesis. An alternative approach to bypass substrate limitations is the assembly of non-native precursor routes, such as the MatB-catalyzed reaction from malonate to malonyl-CoA [28, 75, 93, 185]. The exogenous supply of biosynthetic precursors presupposes a sufficient cellular uptake by passive diffusion and/or dedicated transport systems. If substrate uptake is an issue, one might consider the reconstitution of appropriate uptake system. An illustrative example is the transporter Mae1 from *Schizosaccharomyces pombe*, which can be used to compensate for the lack of malonate uptake in *S. cerevisiae* [149]. A generally applicable option is to produce the PKSs and NRPSs recombinantly in yeast, but to conduct the biosynthesis under in vitro conditions following cell lysis. In this way, transport limitations can be circumvented, as nicely demonstrated in simvastatin biosynthesis [128].
(ii) Phosphopantetheinylation is essential for the activation of NRPSs and PKSs and can be addressed by overexpression of PPTase genes in *S. cerevisiae*. While a clear recommendation for a specific type of PPTase cannot be deduced from the analyzed studies, it seems that the PPTase NpgA from *A. nidulans* is slightly better adapted for the activation of fungal PKSs and NRPSs than its bacterial homolog Sfp [80].
(iii) There are many enzymes other than PKSs and NRPSs, which are involved in the processing and proofreading of biosynthetic intermediates. These tailoring enzymes can play a pivotal role in secondary metabolite biosynthesis and it has become clear that their mere co-expression is often insufficient to achieve high product titers in *S. cerevisiae*. Instead, a balanced expression of tailoring enzymes is necessary, which must also take specific requirements such as subcellular localization, pH optima and cofactor availability into account.
(iv) A universal approach to elevate polyketide and nonribosomal peptide biosynthesis is based on overexpression of PKS and NRPS genes. For this, various strategies were pursued including the usage of strong constitutive promoters, the expression from multicopy (2µ-based) plasmids, or multicopy genomic integration. While overexpression was in general beneficial to increase the product yield, there is no clear evidence, which promoter or multicopy approach is superior. Still, there are only few investigations on how a balanced expression of single pathway enzymes can be used to minimize enzymatic bottlenecks in the concerted product formation of tailoring enzymes and PKS/NRPS assembly lines. Screening of different promoter combinations for all biosynthesis enzymes is a straightforward approach to identify expression conditions that lead to high product titers [48]. Another very useful strategy is to improve the translational efficiency, which is particularly important for large proteins such as PKSs and NRPSs. This can be implemented by codon adaptation, as demonstrated in 6-MSA production [126], or by expressing tRNA genes with anticodon sequences, which are scarce in *S. cerevisiae* [75].

In summary, polyketides and nonribosomal peptides can be heterologously produced in *S. cerevisiae*. The existing literature indicates that it is even possible to exceed the titers of the native producer, depending on the type of biosynthesis enzyme and additional metabolic engineering efforts. It is conceivable that other yeasts with higher biomass yields might be superior to *S. cerevisiae* in terms of polyketide and nonribosomal peptide production, yet there are only few studies addressing this topic [186–191]. Recurring issues that are associated with the heterologous production of PKS- and NRPS-derived metabolites in *S. cerevisiae* have been described in this review together with possible solution strategies. We, the authors, hope that readers will find this review a useful guide for own PKS and NRPS reconstitution experiments in this exciting microbial workhorse.

## Supplementary Information

**Additional file 1**: **Table S1**. Outline of microbial polyketides that were heterologously produced in *S. cerevisiae*, including information on the native producer, the relevant properties of the engineered *S. cerevisiae* strain, the production conditions in yeast and the titer increase compared to the native producer. **Table S2**. Outline of NRPS-derived secondary metabolites that were heterologously produced in *S. cerevisiae*, including information on the native producer, the relevant properties of the engineered *S. cerevisiae* strain, the production conditions in yeast and the titer increase compared to the native producer. **Figure S1**. Integration of polyketide and nonribosomal peptide biosynthesis into the metabolic network of *S. cerevisiae*.

## Data Availability

Data sharing not applicable to this article as no datasets were generated or analyzed during the current study.

## Abbreviations

3Hpcd: 3-Hydroxypropionyl-CoA dehydratase
3Hpcs: 3-Hydroxypropionyl-CoA synthase
6-MOSA: 6-Methyl-orsellinic acid
6-MSA: 6-Methylsalicylic acid
6-MSAS: 6-Methylsalicylic acid synthase
A domain: Adenylation domain
aa: Amino acids
AccA: Biotin carrier protein/biotin carboxylase subunit
ACP: Acyl carrier protein
Acr: Acryloyl-CoA reductase
AibA/B: Glutaconate-CoA transferase
AibC: AibA/B corresponding dehydrogenase
Aro3/Aro4: 3-Deoxy-D-arabino-heptulosonate-7-phosphate synthase
Aro7: Chorismate mutase
AT: Acyltransferase
BktB: β-Ketothiolase
BpsA: Indigoidine synthetase
C domain: Condensation domain
CcrCa: Crotonyl-CoA carboxylase/reductase
C-MeT: C-methylation domain
CoA: Coenzyme A
COM domain: Communication-mediating domain
Crt: Crotonase
DAHP: 3-Deoxy-D-arabino-heptulosonate-7-phosphate
DEBS: Deoxyerythronolide B megasynthase
DH: Dehydratase
DHBA: 2,3-Dihydroxybenzoate
d-Hiv: D-hydroxyisovaleric acid
DMB-SMMP: α-Dimethylbutyryl-S-methyl-mercaptopropionate
DMLA: Dihydromonacolin L acid
E domain: Epimerization domain
E4P: Erythrose-4-phosphate
ER: Enoyl reductase
HR: Highly reducing
HRPKS: Highly-reducing PKS
KIVR: Ketoisovalerate reductase
KR: Ketoreductase
KS: β-Ketoacylsynthase
LDKS (LovF): Lovastatin diketide synthase
LiuC: 3-Methylglutaconyl-CoA hydratase
LNKS (LovB): Lovastatin nonaketide synthase
MatB: Malonyl-CoA synthetase
McrCa: Malonyl-CoA reductase
MJA: Monacolin J acid
MLA: Monacolin L acid
MT: Methyltransferase
NpgA: Phosphopantetheinyl transferase from *Aspergillus nidulans*
NR: Non-reducing
NRPKS: Non-reducing PKS
NRPS: Nonribosomal peptide synthetase
OSA: Orsellinic acid
OSAS: Orsellinic acid synthase
Ox domain: Oxidation domain
PaaH1: 3-Hydroxyacyl-CoA dehydrogenase
PCC: Propionyl-CoA carboxylase
PccB: Transcarboxylase protein/subunit
PccB/AccA: PrpE and PCC complex
PCP: Peptidyl carrier protein
PEP: Phosphoenolpyruvate
PKS: Polyketide synthase
PPTase: Phosphopantetheinyl transferase
PR: Partially reducing
PrpE: Propionyl-CoA synthetase
PRPKS: Partially-reducing PKS
PT domain: Product-template domain
R domain: Reduction domain
Rki1: Ribose-5-phosphate ketolisomerase
SAT: Starter unit AT domain
Sfp: Phosphopantetheinyl transferase from *Bacillus subtilis*
SNAC-ester: *N*-Acetylcysteamine thioester
SrfAC: Surfactin synthetase
Tal1: Transaldolase
TE: Thioesterase
Ter: Trans-enoyl-CoA reductase
Tkl: Transketolase
TKL: Triketide lactone
TycA: Tyrocidine synthetase
Zwf1: Glucose-6-phosphate dehydrogenase

## References

[1] Cox RJ. Polyketides, proteins and genes in fungi: programmed nano-machines begin to reveal their secrets. Org Biomol Chem. 2007;5:2010. www.rsc.org/obc 10.1039/b704420h 17581644

[2] Süssmuth RD, Mainz A (2017). Nonribosomal peptide synthesis-principles and prospects. Angew Chemie Int Ed.

[3] Hertweck C (2009). The biosynthetic logic of polyketide diversity. Angew Chemie Int Ed.

[4] Newman DJ, Cragg GM (2020). Natural products as sources of new drugs over the nearly four decades from 01/1981 to 09/2019. J Nat Prod.

[5] Reisman SE, Maimone TJ (2021). Total synthesis of complex natural products: more than a race for molecular summits. Acc Chem Res.

[6] Sheldon RA, Woodley JM. Role of biocatalysis in sustainable chemistry. Chem Rev 2018;118:801–38. https://pubmed.ncbi.nlm.nih.gov/28876904/10.1021/acs.chemrev.7b00203 28876904

[7] Siddiqui MS, Thodey K, Trenchard I, Smolke CD (2012). Advancing secondary metabolite biosynthesis in yeast with synthetic biology tools. FEMS Yeast Res.

[8] Rohr J. Cryptophycin anticancer drugs revisited. ACS Chem Biol 2006;1:747–50. www.acschemicalbiology.org 10.1021/cb6004678 17240971

[9] Mitrović I, Lukić N, Grahovac M, Jokić A, Dodić J, Grahovac J (2021). Optimization of streptomyces hygroscopicus cultivation parameters in a lab-scale bioreactor. Chem Eng Technol.

[10] Lubertozzi D, Keasling JD. Developing Aspergillus as a host for heterologous expression. Biotechnol Adv 2009;27:53–75. https://linkinghub.elsevier.com/retrieve/pii/S0734975008000906 10.1016/j.biotechadv.2008.09.001 18840517

[11] Gómez S, Fernández FJ, Vega MC. Heterologous Expression of Proteins in Aspergillus. In: Gupta VK, editor. New and future developments in microbial biotechnology and bioengineering: Aspergillus system properties and applications*.* Elsevier; 2016. p. 55–68. https://linkinghub.elsevier.com/retrieve/pii/B978044463505100004X

[12] Pfeifer BA, Khosla C. Biosynthesis of polyketides in heterologous hosts. Microbiol Mol Biol Rev 2001;65:106–18. http://mmbr.asm.org/10.1128/MMBR.65.1.106-118.2001 PMC99020 11238987

[13] Zhang JJ, Tang X, Moore BS (2019). Genetic platforms for heterologous expression of microbial natural products. Nat Prod Rep.

[14] Lee NCO, Larionov V, Kouprina N. Highly efficient CRISPR/Cas9-mediated TAR cloning of genes and chromosomal loci from complex genomes in yeast. Nucleic Acids Res 2015;43:e55–e55. http://www.ncbi.nlm.nih.gov/pubmed/25690893 10.1093/nar/gkv112 PMC4417148 25690893

[15] Kouprina N, Larionov V. TAR cloning: insights into gene function, long-range haplotypes and genome structure and evolution. Nat Rev Genet 2006;7:805–12. www.nature.com/reviews/genetics 10.1038/nrg1943 16983376

[16] Larionov V, Kouprina N, Graves J, Chen XN, Korenberg JR, Resnick MA. Specific cloning of human DNA as yeast artificial chromosomes by transformation-associated recombination. Proc Natl Acad Sci 1996;93:491–6. https://pubmed.ncbi.nlm.nih.gov/8552668/10.1073/pnas.93.1.491 PMC40264 8552668

[17] Zhang B, Tian W, Wang S, Yan X, Jia X, Pierens GK (2017). Activation of natural products biosynthetic pathways via a protein modification level regulation. ACS Chem Biol.

[18] Zhang JJ, Yamanaka K, Tang X, Moore BS (2019). Direct cloning and heterologous expression of natural product biosynthetic gene clusters by transformation-associated recombination. Methods Enzymol.

[19] Bai Flagfeldt D, Siewers V, Huang L, Nielsen J (2009). Characterization of chromosomal integration sites for heterologous gene expression in* Saccharomyces cerevisiae*. Yeast.

[20] Maury J, Germann SM, Baallal Jacobsen SA, Jensen NB, Kildegaard KR, Herrgård MJ, et al. EasyCloneMulti: a set of vectors for simultaneous and multiple genomic integrations in *Saccharomyces cerevisiae*. Isalan M, editor. PLoS One 2016;11:e0150394. http://www.ncbi.nlm.nih.gov/pubmed/26934490 10.1371/journal.pone.0150394 PMC4775045 26934490

[21] Horwitz AA, Walter JM, Schubert MG, Kung SH, Hawkins K, Platt DM, et al. Efficient multiplexed integration of synergistic alleles and metabolic pathways in yeasts via CRISPR-Cas. Cell Syst 2015; 1:88–96. https://www.sciencedirect.com/science/article/pii/S2405471215000034?via%3Dihub 10.1016/j.cels.2015.02.001 27135688

[22] Reider Apel A, D’Espaux L, Wehrs M, Sachs D, Li RA, Tong GJ (2017). A Cas9-based toolkit to program gene expression in *Saccharomyces cerevisiae*. Nucleic Acids Res.

[23] DiCarlo JE, Norville JE, Mali P, Rios X, Aach J, Church GM. Genome engineering in* Saccharomyces cerevisiae* using CRISPR-Cas systems. Nucleic Acids Res 2013;41:4336–43. http://www.ncbi.nlm.nih.gov/pubmed/23460208 10.1093/nar/gkt135 PMC3627607 23460208

[24] Blount BA, Gowers G-OF, Ho JCH, Ledesma-Amaro R, Jovicevic D, McKiernan RM, et al. Rapid host strain improvement by in vivo rearrangement of a synthetic yeast chromosome. Nat Commun 2018;9:1932. http://www.nature.com/articles/s41467-018-03143-w 10.1038/s41467-018-03143-w PMC5964169 29789540

[25] Yamanaka K, Reynolds KA, Kersten RD, Ryan KS, Gonzalez DJ, Nizet V (2014). Direct cloning and refactoring of a silent lipopeptide biosynthetic gene cluster yields the antibiotic taromycin A. Proc Natl Acad Sci U S A.

[26] Lee ME, DeLoache WC, Cervantes B, Dueber JE (2015). A highly characterized yeast toolkit for modular, multipart assembly. ACS Synth Biol.

[27] Yuan J, Ching CB. Combinatorial assembly of large biochemical pathways into yeast chromosomes for improved production of value-added compounds. ACS Synth Biol 2015;4:23–31. https://pubs.acs.org/sharingguidelines 10.1021/sb500079f 24847678

[28] Tsunematsu Y, Ishiuchi K, Hotta K, Watanabe K. Yeast-based genome mining, production and mechanistic studies of the biosynthesis of fungal polyketide and peptide natural products. Nat Prod Rep 2013;30:1139–49. www.rsc.org/npr 10.1039/c3np70037b 23824111

[29] Shao Z, Zhao H, Zhao H (2009). DNA assembler, an in vivo genetic method for rapid construction of biochemical pathways. Nucleic Acids Res.

[30] Shao Z, Luo Y, Zhao H. Rapid characterization and engineering of natural product biosynthetic pathways via DNA assembler. Mol Biosyst 2011;7:1056. www.rsc.org/molecularbiosystems 10.1039/c0mb00338g PMC4320646 21327279

[31] Jansen G, Wu C, Schade B, Thomas DY, Whiteway M. Drag & drop cloning in yeast. Gene 2005;344:43–51. https://linkinghub.elsevier.com/retrieve/pii/S0378111904006420 10.1016/j.gene.2004.10.016 15656971

[32] Naesby M, Nielsen SV, Nielsen CAF, Green T, Tange TO, Simón E, et al. Yeast artificial chromosomes employed for random assembly of biosynthetic pathways and production of diverse compounds in* Saccharomyces cerevisiae*. Microb Cell Fact 2009;8:45. https://pubmed.ncbi.nlm.nih.gov/19678954/10.1186/1475-2859-8-45 PMC2732597 19678954

[33] Li A, Liu Z, Li Q, Yu L, Wang D, Deng X (2008). Construction and characterization of bidirectional expression vectors in *Saccharomyces cerevisiae*. FEMS Yeast Res.

[34] Öztürk S, Ergün BG, Çalık P (2017). Double promoter expression systems for recombinant protein production by industrial microorganisms. Appl Microbiol Biotechnol.

[35] Curran KA, Morse NJ, Markham KA, Wagman AM, Gupta A, Alper HS (2015). Short synthetic terminators for improved heterologous gene expression in yeast. ACS Synth Biol.

[36] Redden H, Alper HS. The development and characterization of synthetic minimal yeast promoters. Nat Commun 2015;6:7810. http://www.ncbi.nlm.nih.gov/pubmed/26183606 10.1038/ncomms8810 PMC4518256 26183606

[37] Blazeck J, Garg R, Reed B, Alper HS (2012). Controlling promoter strength and regulation in *Saccharomyces cerevisiae* using synthetic hybrid promoters. Biotechnol Bioeng.

[38] Singh A, Lugovoy JM, Kohr WJ, Perry LJ (1984). Synthesis, secretion and processing of alpha-factor-interferon fusion proteins in yeast. Nucleic Acids Res.

[39] Kjeldsen T (2000). Yeast secretory expression of insulin precursors. Appl Microbiol Biotechnol.

[40] Jan Markussen, Ulrik Damgaard, Ivan Diers, Niels Pill, Mogens Trier Hansen, Per Larsen, Fanny Norris, Kjeld Norris, Ole Schou, Leo Snel, Lars Thim HOV. Porto Carras, Chalkidiki, Greece, Aug. 31–Sept. 5, 1986 Theodoropoulos D, editor. Porto Carras, Chalkidiki, Greece, Aug. 31–Sept. 5, 1986. Berlin: De Gruyter; 1987. Doi: 10.1515/9783110864243/html

[41] Brake AJ, Merryweather JP, Coit DG, Heberlein UA, Masiarz FR, Mullenbach GT (1984). Alpha-factor-directed synthesis and secretion of mature foreign proteins in* Saccharomyces cerevisiae*. Proc Natl Acad Sci U S A.

[42] Kim I-K, Roldão A, Siewers V, Nielsen J (2012). A systems-level approach for metabolic engineering of yeast cell factories. FEMS Yeast Res.

[43] Valenzuela P, Medina A, Rutter WJ, Ammerer G, Hall BD. Synthesis and assembly of hepatitis B virus surface antigen particles in yeast. Nature 1982;298:347–50. https://www.nature.com/articles/298347a0 10.1038/298347a0 7045698

[44] Kumar R, Kumar P. Yeast-based vaccines: New perspective in vaccine development and application. FEMS Yeast Res 2019;19:7. http://orcid.org/0000-0003-1922-0030 10.1093/femsyr/foz007 30668686

[45] Porro D, Sauer M, Branduardi P, Mattanovich D (2005). Recombinant protein production in yeasts. Mol Biotechnol.

[46] Kowald A, Wierling C (2011). Standards, tools, and databases for the analysis of yeast ‘omics data. Methods Mol Biol.

[47] Bond C, Tang Y, Li L.* Saccharomyces cerevisiae* as a tool for mining, studying and engineering fungal polyketide synthases. Fungal Genet Biol 2016;89:52–61. https://linkinghub.elsevier.com/retrieve/pii/S1087184516300044 10.1016/j.fgb.2016.01.005 PMC4789138 26850128

[48] Awan AR, Blount BA, Bell DJ, Shaw WM, Ho JCH, McKiernan RM, et al. Biosynthesis of the antibiotic nonribosomal peptide penicillin in baker’s yeast. Nat Commun 2017;8:15202. www.nature.com/naturecommunications 10.1038/ncomms15202 PMC5418595 28469278

[49] Awan AR, Shaw WM, Ellis T (2016). Biosynthesis of therapeutic natural products using synthetic biology. Adv Drug Deliv Rev.

[50] Siddiqui MS, Thodey K, Trenchard I, Smolke CD. Advancing secondary metabolite biosynthesis in yeast with synthetic biology tools. FEMS Yeast Res 2012;12:144–70. https://academic.oup.com/femsyr/article/12/2/144/526081 10.1111/j.1567-1364.2011.00774.x 22136110

[51] Walker RSK, Pretorius IS. Applications of yeast synthetic biology geared towards the production of biopharmaceuticals. Genes (Basel) 2018;9:340. www.mdpi.com/journal/genes 10.3390/genes9070340 PMC6070867 29986380

[52] Nevoigt E. Progress in metabolic engineering of *Saccharomyces cerevisiae*. Microbiol Mol Biol Rev 2008;72:379–412. http://mips.gsf.de/genre/proj/yeast/10.1128/MMBR.00025-07 PMC2546860 18772282

[53] Sauer M, Porro D, Mattanovich D, Branduardi P. 16 years research on lactic acid production with yeast—ready for the market? Biotechnol Genet Eng Rev 2010;27:229–56. https://pubmed.ncbi.nlm.nih.gov/21415900/10.1080/02648725.2010.10648152 21415900

[54] Miller C, Fosmer A, Rush B, McMullin T, Beacom D, Suominen P. Industrial production of lactic acid. Compr Biotechnol 2011: 179–88. https://linkinghub.elsevier.com/retrieve/pii/B978008088504900177X

[55] Rush B. Turning a novel yeast into a platform host for industrial production of fuels and chemicals. Metab Eng IX 2013. Available from: https://dc.engconfintl.org/metabolic_ix/6

[56] Rahmat E, Kang Y (2020). Yeast metabolic engineering for the production of pharmaceutically important secondary metabolites. Appl Microbiol Biotechnol.

[57] Vassaux A, Meunier L, Vandenbol M, Baurain D, Fickers P, Jacques P, et al. Nonribosomal peptides in fungal cell factories: from genome mining to optimized heterologous production. Biotechnol Adv 2019;37:107449. https://pubmed.ncbi.nlm.nih.gov/31518630/10.1016/j.biotechadv.2019.107449 31518630

[58] Ray L, Moore BS. Recent advances in the biosynthesis of unusual polyketide synthase substrates. Nat Prod Rep 2016;33:150–61. www.rsc.org/npr 10.1039/c5np00112a PMC4742410 26571143

[59] Wang J, Zhang R, Chen X, Sun X, Yan Y, Shen X (2020). Biosynthesis of aromatic polyketides in microorganisms using type II polyketide synthases. Microb Cell Fact.

[60] Shimizu Y, Ogata H, Goto S (2017). Type III polyketide synthases: functional classification and phylogenomics. ChemBioChem.

[61] Herbst DA, Townsend CA, Maier T. The architectures of iterative type I PKS and FAS. Nat Prod Rep 2018;35:1046–69. https://pubs.rsc.org/en/content/articlehtml/2018/np/c8np00039e 10.1039/c8np00039e PMC6192843 30137093

[62] van den Berg MA, Westerlaken I, Leeflang C, Kerkman R, Bovenberg RAL. Functional characterization of the penicillin biosynthetic gene cluster of Penicillium chrysogenum Wisconsin54-1255. Fungal Genet Biol 2007;44:830–44. https://pubmed.ncbi.nlm.nih.gov/17548217/10.1016/j.fgb.2007.03.008 17548217

[63] Partida-Martinez LP, de Looss CF, Ishida K, Ishida M, Roth M, Buder K, et al. Rhizonin, the first mycotoxin isolated from the zygomycota, is not a fungal metabolite but is produced by bacterial endosymbionts. Appl Environ Microbiol 2007;73:793–7. /pmc/articles/PMC1800748/10.1128/AEM.01784-06 PMC1800748 17122400

[64] Reimer JM, Haque AS, Tarry MJ, Schmeing TM. Piecing together nonribosomal peptide synthesis. Curr Opin Struct Biol 2018;49:104–13. https://pubmed.ncbi.nlm.nih.gov/29444491/10.1016/j.sbi.2018.01.011 29444491

[65] Finking R, Marahiel MA. Biosynthesis of nonribosomal peptides. Annu Rev Microbiol 2004;58:453–88. www.annualreviews.org 10.1146/annurev.micro.58.030603.123615 15487945

[66] Strieker M, Tanović A, Marahiel MA. Nonribosomal peptide synthetases: structures and dynamics. Curr Opin Struct Biol 2010;20:234–40. https://linkinghub.elsevier.com/retrieve/pii/S0959440X10000126 10.1016/j.sbi.2010.01.009 20153164

[67] May JJ, Kessler N, Marahiel MA, Stubbs MT (2002). Crystal structure of DhbE, an archetype for aryl acid activating domains of modular nonribosomal peptide synthetases. Proc Natl Acad Sci U S A.

[68] Miyanaga A, Kudo F, Eguchi T. Protein-protein interactions in polyketide synthase-nonribosomal peptide synthetase hybrid assembly lines. Nat Prod Rep 2018;35:1185–209. https://pubs.rsc.org/en/content/articlehtml/2018/np/c8np00022k 10.1039/c8np00022k 30074030

[69] Rath CM, Scaglione JB, Kittendorf JD, Sherman DH. NRPS/PKS Hybrid Enzymes and Their Natural Products. Compr Nat Prod II Chem Biol 2010: 453–92. https://linkinghub.elsevier.com/retrieve/pii/B9780080453828007255

[70] Fisch KM. Biosynthesis of natural products by microbial iterative hybrid PKS–NRPS. RSC Adv 2013;3:18228. www.rsc.org/advances

[71] Boettger D, Hertweck C. Molecular diversity sculpted by fungal PKS-NRPS hybrids. Chembiochem 2013;14:28–42. https://pubmed.ncbi.nlm.nih.gov/23225733/10.1002/cbic.201200624 23225733

[72] Mofid MR, Finking R, Essen LO, Marahiel MA (2004). Structure-based mutational analysis of the 4‘-phosphopantetheinyl transferases Sfp from *Bacillus subtilis*: carrier protein recognition and reaction mechanism. Biochemistry.

[73] Copp JN, Neilan BA (2006). The phosphopantetheinyl transferase superfamily: phylogenetic analysis and functional implications in cyanobacteria. Appl Environ Microbiol.

[74] Beld J, Sonnenschein EC, Vickery CR, Noel JP, Burkart MD. The phosphopantetheinyl transferases: catalysis of a post-translational modification crucial for life. Nat Prod Rep 2014;31:61–108. http://xlink.rsc.org/?DOI=C3NP70054B 10.1039/c3np70054b PMC3918677 24292120

[75] Mutka SC, Bondi SM, Carney JR, Da Silva NA, Kealey JT. Metabolic pathway engineering for complex polyketide biosynthesis in *Saccharomyces cerevisiae*. FEMS Yeast Res 2006;6:40–7. http://www.ncbi.nlm.nih.gov/pubmed/16423069 10.1111/j.1567-1356.2005.00001.x 16423069

[76] Gao X, Haynes SW, Ames BD, Wang P, Vien LP, Walsh CT, et al. Cyclization of fungal nonribosomal peptides by a terminal condensation-like domain. Nat Chem Biol 2012;8:823–30. http://www.nature.com/articles/nchembio.1047 10.1038/nchembio.1047 PMC3505271 22902615

[77] Reuter K, Mofid MR, Marahiel MA, Ficner R (1999). Crystal structure of the surfactin synthetase-activating enzyme sfp: a prototype of the 4’-phosphopantetheinyl transferase superfamily. EMBO J.

[78] Sánchez C, Du L, Edwards DJ, Toney MD, Shen B (2001). Cloning and characterization of a phosphopantetheinyl transferase from Streptomyces verticillus ATCC15003, the producer of the hybrid peptide-polyketide antitumor drug bleomycin. Chem Biol Cell Press.

[79] Keszenman-Pereyra D, Lawrence S, Twfieg M-E, Price J, Turner G. The npgA/ cfwA gene encodes a putative 4’-phosphopantetheinyl transferase which is essential for penicillin biosynthesis in* Aspergillus nidulans*. Curr Genet 2003;43:186–90. www.tigr.org/tdb/e2k1/afu1/10.1007/s00294-003-0382-7 12664133

[80] Wattanachaisaereekul S, Lantz AE, Nielsen ML, Andrésson ÓS, Nielsen J (2007). Optimization of heterologous production of the polyketide 6-MSA in *Saccharomyces cerevisiae*. Biotechnol Bioeng.

[81] Kealey JT, Liu L, Santi DV, Betlach MC, Barr PJ (1998). Production of a polyketide natural product in nonpolyketide-producing prokaryotic and eukaryotic hosts. Proc Natl Acad Sci U S A.

[82] Wehrs M, Prahl J-P, Moon J, Li Y, Tanjore D, Keasling JD (2019). Correction to: Production efficiency of the bacterial non-ribosomal peptide indigoidine relies on the respiratory metabolic state in *S. cerevisiae*. Microb Cell Fact.

[83] Henritzi S, Fischer M, Grininger M, Oreb M, Boles E (2018). An engineered fatty acid synthase combined with a carboxylic acid reductase enables de novo production of 1-octanol in *Saccharomyces cerevisiae*. Biotechnol Biofuels.

[84] Chen Y, Daviet L, Schalk M, Siewers V, Nielsen J. Establishing a platform cell factory through engineering of yeast acetyl-CoA metabolism. Metab Eng 2013;15:48–54. https://pubmed.ncbi.nlm.nih.gov/23164578/10.1016/j.ymben.2012.11.002 23164578

[85] Lian J, Si T, Nair NU, Zhao H. Design and construction of acetyl-CoA overproducing *Saccharomyces cerevisiae* strains. Metab Eng 2014;24:139–49. http://www.ncbi.nlm.nih.gov/pubmed/24853351 10.1016/j.ymben.2014.05.010 24853351

[86] Hitschler J, Grininger M, Boles E. Substrate promiscuity of polyketide synthase enables production of tsetse fly attractants 3-ethylphenol and 3-propylphenol by engineering precursor supply in yeast. Sci Rep 2020;10:9962. www.nature.com/scientificreports 10.1038/s41598-020-66997-5 PMC7305150 32561880

[87] Wattanachaisaereekul S, Lantz AE, Nielsen ML, Nielsen J. Production of the polyketide 6-MSA in yeast engineered for increased malonyl-CoA supply. Metab Eng 2008;10:246–54. https://pubmed.ncbi.nlm.nih.gov/18555717/10.1016/j.ymben.2008.04.005 18555717

[88] Luttik MA, Kötter P, Salomons FA, van der Klei IJ, van Dijken JP, Pronk JT. The* Saccharomyces cerevisiae* ICL2 gene encodes a mitochondrial 2-methylisocitrate lyase involved in propionyl-coenzyme A metabolism. J Bacteriol 2000;182:7007–13. http://www.rz.uni-frankfurt.de/FB 10.1128/jb.182.24.7007-7013.2000 PMC94827 11092862

[89] Pronk JT, van der Linden-Beuman A, Verduyn C, Scheffers WA, van Dijken JP (1994). Propionate metabolism in *Saccharomyces cerevisiae*: implications for the metabolon hypothesis. Microbiology.

[90] Krink-Koutsoubelis N, Loechner AC, Lechner A, Link H, Denby CM, Vögeli B, et al. Engineered production of short-chain acyl-coenzyme a esters in *Saccharomyces cerevisiae*. ACS Synth Biol 2018;7:1105–15. https://escholarship.org/uc/item/0ms113jv 10.1021/acssynbio.7b00466 29498824

[91] Choi JW, Da Silva NA. Improving polyketide and fatty acid synthesis by engineering of the yeast acetyl-CoA carboxylase. J Biotechnol 2014;187:56–9. http://www.ncbi.nlm.nih.gov/pubmed/25078432 10.1016/j.jbiotec.2014.07.430 25078432

[92] Shi S, Chen Y, Siewers V, Nielsen J. Improving production of malonyl coenzyme A-derived metabolites by abolishing Snf1-dependent regulation of Acc1. MBio 2014;5. http://mbio.asm.org/10.1128/mBio.01130-14 PMC4010835 24803522

[93] Ishiuchi K, Nakazawa T, Ookuma T, Sugimoto S, Sato M, Tsunematsu Y (2012). Establishing a new methodology for genome mining and biosynthesis of polyketides and peptides through yeast molecular genetics. ChemBioChem.

[94] Schadeweg V, Boles E. n-Butanol production in* Saccharomyces cerevisiae* is limited by the availability of coenzyme A and cytosolic acetyl-CoA. Biotechnol Biofuels 2016;9:44. http://www.biotechnologyforbiofuels.com/content/9/1/44 10.1186/s13068-016-0456-7 PMC4765181 26913077

[95] Schadeweg V, Boles E (2016). Increasing n-butanol production with* Saccharomyces cerevisiae* by optimizing acetyl-CoA synthesis, NADH levels and trans-2-enoyl-CoA reductase expression. Biotechnol Biofuels.

[96] Cardenas J, Da Silva NA (2016). Engineering cofactor and transport mechanisms in *Saccharomyces cerevisiae* for enhanced acetyl-CoA and polyketide biosynthesis. Metab Eng.

[97] Van Hoek P, Van Dijken JP, Pronk JT. Effect of specific growth rate on fermentative capacity of baker’s yeast. Appl Environ Microbiol 1998;64:4226–33. http://www.ncbi.nlm.nih.gov/pubmed/9797269 10.1128/aem.64.11.4226-4233.1998 PMC106631 9797269

[98] Vemuri GN, Eiteman MA, McEwen JE, Olsson L, Nielsen J (2007). Increasing NADH oxidation reduces overflow metabolism in *Saccharomyces cerevisiae*. Proc Natl Acad Sci U S A.

[99] Jessop-Fabre MM, Dahlin J, Biron MB, Stovicek V, Ebert BE, Blank LM, et al. The Transcriptome and flux profiling of crabtree-negative hydroxy acid-producing strains of *Saccharomyces cerevisiae* reveals changes in the central carbon metabolism. Biotechnol J 2019;14:e1900013. https://pubmed.ncbi.nlm.nih.gov/30969019/10.1002/biot.201900013 30969019

[100] Averesch NJH, Krömer JO. Metabolic engineering of the shikimate pathway for production of aromatics and derived compounds—present and future strain construction strategies. Front Bioeng Biotechnol 2018;6:32. www.frontiersin.org 10.3389/fbioe.2018.00032 PMC5879953 29632862

[101] de Mattos-Shipley KMJ, Greco C, Heard DM, Hough G, Mulholland NP, Vincent JL, et al. The cycloaspeptides: uncovering a new model for methylated nonribosomal peptide biosynthesis. Chem Sci 2018;9:4109–17. https://pubs.rsc.org/en/content/articlehtml/2018/sc/c8sc00717a 10.1039/c8sc00717a PMC5941284 29780540

[102] Korp J, König S, Schieferdecker S, Dahse H-M, König GM, Werz O, et al. Harnessing enzymatic promiscuity in myxochelin biosynthesis for the production of 5-lipoxygenase inhibitors. Chembiochem 2015;16:2445–50. http://www.ncbi.nlm.nih.gov/pubmed/26416255 10.1002/cbic.201500446 26416255

[103] Lingens F, Goebel W, Uesseler H. Regulation der Biosynthese der aromatischen Aminosäuren in *Saccharomyces cerevisiae*. In: Liébecq C, editor. European Journal of Biochemistry, 1st edn. Berlin, Heidelberg: Springer Berlin Heidelberg; 1967. p. 363–74. Doi: 10.1007/978-3-662-25813-2_50 10.1111/j.1432-1033.1967.tb00083.x 6060189

[104] Luttik MAH, Vuralhan Z, Suir E, Braus GH, Pronk JT, Daran JM. Alleviation of feedback inhibition in* Saccharomyces cerevisiae* aromatic amino acid biosynthesis: quantification of metabolic impact. Metab Eng 2008;10:141–53. https://pubmed.ncbi.nlm.nih.gov/18372204/10.1016/j.ymben.2008.02.002 18372204

[105] Liu Q, Yu T, Li X, Chen Y, Campbell K, Nielsen J, et al. Rewiring carbon metabolism in yeast for high level production of aromatic chemicals. Nat Commun 2019;10:4976. http://www.nature.com/articles/s41467-019-12961-5 10.1038/s41467-019-12961-5 PMC6823513 31672987

[106] Mao J, Liu Q, Song X, Wang H, Feng H, Xu H (2017). Combinatorial analysis of enzymatic bottlenecks of L-tyrosine pathway by p-coumaric acid production in *Saccharomyces cerevisiae*. Biotechnol Lett.

[107] Gold ND, Gowen CM, Lussier F-X, Cautha SC, Mahadevan R, Martin VJJ (2015). Metabolic engineering of a tyrosine-overproducing yeast platform using targeted metabolomics. Microb Cell Fact.

[108] Kallscheuer N, Kage H, Milke L, Nett M, Marienhagen J (2019). Microbial synthesis of the type I polyketide 6-methylsalicylate with Corynebacterium glutamicum. Appl Microbiol Biotechnol.

[109] Li Y, Chooi Y-H, Sheng Y, Valentine JS, Tang Y. Comparative characterization of fungal anthracenone and naphthacenedione biosynthetic pathways reveals an α-hydroxylation-dependent Claisen-like cyclization catalyzed by a dimanganese thioesterase. J Am Chem Soc 2011;133:15773–85. https://pubs.acs.org/sharingguidelines 10.1021/ja206906d PMC3183131 21866960

[110] Sun L. Investigation and engineering of polyketide biosynthetic pathways. All Grad. Theses Diss. 6903. Utah State University; 2017. https://digitalcommons.usu.edu/etd/6903

[111] Jakočiūnas T, Klitgaard AK, Kontou EE, Nielsen JB, Thomsen E, Romero-Suarez D, et al. Programmable polyketide biosynthesis platform for production of aromatic compounds in yeast. Synth Syst Biotechnol 2020;5:11–8. https://linkinghub.elsevier.com/retrieve/pii/S2405805X20300041 10.1016/j.synbio.2020.01.004 PMC6992897 32021916

[112] Rugbjerg P, Naesby M, Mortensen UH, Frandsen RJN (2013). Reconstruction of the biosynthetic pathway for the core fungal polyketide scaffold rubrofusarin in* Saccharomyces cerevisiae*. Microb Cell Fact.

[113] Zabala AO, Chooi Y-H, Choi MS, Lin H-C, Tang Y. Fungal polyketide synthase product chain-length control by partnering thiohydrolase. ACS Chem Biol 2014;9:1576–86. https://pubs.acs.org/sharingguidelines 10.1021/cb500284t PMC4215887 24845309

[114] Zhou H, Qiao K, Gao Z, Vederas JC, Tang Y. Insights into radicicol biosynthesis via heterologous synthesis of intermediates and analogs. J Biol Chem 2010;285:41412–21. http://www.ncbi.nlm.nih.gov/pubmed/20961859 10.1074/jbc.M110.183574 PMC3009867 20961859

[115] Reeves CD, Hu Z, Reid R, Kealey JT. Genes for the biosynthesis of the fungal polyketides hypothemycin from *Hypomyces subiculosus* and radicicol from *Pochonia chlamydosporia*. Appl Environ Microbiol 2008;74:5121–9. http://aem.asm.org/10.1128/AEM.00478-08 PMC2519290 18567690

[116] Zhou H, Qiao K, Gao Z, Meehan MJ, Li JWH, Zhao X, et al. Enzymatic synthesis of resorcylic acid lactones by cooperation of fungal iterative polyketide synthases involved in hypothemycin biosynthesis. J Am Chem Soc 2010;132:4530–1. http://pubs.acs.org. 10.1021/ja100060k PMC2861853 20222707

[117] Cochrane RVK, Gao Z, Lambkin GR, Xu W, Winter JM, Marcus SL (2015). Comparison of 10,11-dehydrocurvularin polyketide synthases from Alternaria cinerariae and Aspergillus terreus highlights key structural motifs. ChemBioChem.

[118] Xu Y, Zhou T, Zhang S, Espinosa-Artiles P, Wang L, Zhang W (2014). Diversity-oriented combinatorial biosynthesis of benzenediol lactone scaffolds by subunit shuffling of fungal polyketide synthases. Proc Natl Acad Sci U S A.

[119] Xu Y, Zhou T, Espinosa-Artiles P, Tang Y, Zhan J, Molnár I. Insights into the biosynthesis of 12-membered resorcylic acid lactones from heterologous production in* Saccharomyces cerevisiae*. ACS Chem Biol 2014;9:1119–27. https://pubmed.ncbi.nlm.nih.gov/24597618/10.1021/cb500043g PMC4033647 24597618

[120] Xu Y, Espinosa-Artiles P, Schubert V, Xu Y ming, Zhang W, Lin M, et al. Characterization of the biosynthetic genes for 10,11- dehydrocurvularin, a heat shock response-modulating anticancer fungal polyketide from Aspergillus terreus. Appl Environ Microbiol 2013;79:2038–47. https://pubmed.ncbi.nlm.nih.gov/23335766/10.1128/AEM.03334-12 PMC3592213 23335766

[121] Zhao M, Zhao Y, Yao M, Iqbal H, Hu Q, Liu H (2020). Pathway engineering in yeast for synthesizing the complex polyketide bikaverin. Nat Commun.

[122] Chooi Y-H, Krill C, Barrow RA, Chen S, Trengove R, Oliver RP, et al. An in planta-expressed polyketide synthase produces (R)-mellein in the wheat pathogen Parastagonospora nodorum. Appl Environ Microbiol 2015;81:177–86. http://www.ncbi.nlm.nih.gov/pubmed/25326302 10.1128/AEM.02745-14 PMC4272741 25326302

[123] Winter JM, Sato M, Sugimoto S, Chiou G, Garg NK, Tang Y, et al. Identification and characterization of the chaetoviridin and chaetomugilin gene cluster in Chaetomium globosum reveal dual functions of an iterative highly-reducing polyketide synthase. J Am Chem Soc 2012;134:17900–3. https://pubs.acs.org/sharingguidelines 10.1021/ja3090498 PMC3494086 23072467

[124] Winter JM, Chiou G, Bothwell IR, Xu W, Garg NK, Luo M, et al. Expanding the structural diversity of polyketides by exploring the cofactor tolerance of an inline methyltransferase domain. Org Lett 2013;15:3774–7. https://pubs.acs.org/sharingguidelines 10.1021/ol401723h PMC3779521 23837609

[125] Winter JM, Cascio D, Dietrich D, Sato M, Watanabe K, Sawaya MR, et al. Biochemical and structural basis for controlling chemical modularity in fungal polyketide biosynthesis. J Am Chem Soc 2015;137:9885–93. https://pubs.acs.org/sharingguidelines 10.1021/jacs.5b04520 PMC4922798 26172141

[126] Hitschler J, Boles E. De novo production of aromatic m-cresol in* Saccharomyces cerevisiae* mediated by heterologous polyketide synthases combined with a 6-methylsalicylic acid decarboxylase. Metab Eng Commun 2019;9:e00093. https://linkinghub.elsevier.com/retrieve/pii/S2214030119300082 10.1016/j.mec.2019.e00093 PMC6520567 31193192

[127] Wang M, Beissner M, Zhao H (2014). Aryl-aldehyde formation in fungal polyketides: discovery and characterization of a distinct biosynthetic mechanism. Chem Biol.

[128] Bond CM, Tang Y. Engineering *Saccharomyces cerevisiae* for production of simvastatin. Metab Eng 2019;51:1–8. https://linkinghub.elsevier.com/retrieve/pii/S1096717618302349 10.1016/j.ymben.2018.09.005 PMC6348118 30213650

[129] Ma SM, Li JWH, Choi JW, Zhou H, Lee KKM, Moorthie VA, et al. Complete reconstitution of a highly reducing iterative polyketide synthase. Science 2009;326:589–92. www.sciencemag.org/cgi/content/full/1179052/DC1 10.1126/science.1175602 PMC2875069 19900898

[130] Xu W, Chooi Y-H, Choi JW, Li S, Vederas JC, Da Silva NA (2013). LovG: the thioesterase required for dihydromonacolin L release and lovastatin nonaketide synthase turnover in lovastatin biosynthesis. Angew Chem Int Ed Engl.

[131] Ryan SD, Harris CS, Mo F, Lee H, Hou ST, Bazan NG, et al. Platelet activating factor-induced neuronal apoptosis is initiated independently of its G-protein coupled PAF receptor and is inhibited by the benzoate orsellinic acid. J Neurochem 2007;103:88–97. https://pubmed.ncbi.nlm.nih.gov/17877634/10.1111/j.1471-4159.2007.04740.x 17877634

[132] Lopes TIB, Coelho RG, Yoshida NC, Honda NK. Radical-scavenging activity of orsellinates. Chem Pharm Bull (Tokyo) [Internet]. Chem Pharm Bull (Tokyo); 2008 [cited 2020 Nov 24];56:1551–4. Available from: https://pubmed.ncbi.nlm.nih.gov/18981604/10.1248/cpb.56.1551 18981604

[133] National Center for Advancing Translational Sciences. Inxight Drugs. US Dep. Heal. Hum. Serv. 2017. https://drugs.ncats.io/substances?facet=Originator%2FAndre, G.

[134] Müller K. Pharmaceutically relevant metabolites from lichens. Appl Microbiol Biotechnol 2001;56:9–16. https://pubmed.ncbi.nlm.nih.gov/11499952/10.1007/s002530100684 11499952

[135] Sanchez JF, Chiang Y-M, Szewczyk E, Davidson AD, Ahuja M, Elizabeth Oakley C, et al. Molecular genetic analysis of the orsellinic acid/F9775 gene cluster of* Aspergillus nidulans*. Mol Biosyst 2010;6:587–93. http://www.ncbi.nlm.nih.gov/pubmed/20174687 10.1039/b904541d PMC2903553 20174687

[136] Lackner G, Bohnert M, Wick J, Hoffmeister D (2013). Assembly of melleolide antibiotics involves a polyketide synthase with cross-coupling activity. Chem Biol.

[137] Gaucher GM, Shepherd MG. Isolation of orsellinic acid synthase. Biochem Biophys Res Commun 1968;32:664–71. https://pubmed.ncbi.nlm.nih.gov/5682289/10.1016/0006-291x(68)90290-8 5682289

[138] BIRKINSHAW JH, GOWLLAND A. Studies in the biochemistry of micro-organisms. 110. Production and biosynthesis of orsellinic acid by Penicillium madriti G. Smith. Biochem J 1962;84:342–7. https://portlandpress.com/biochemj/article/84/2/342/53358/Studies-in-the-biochemistry-of-microorganisms-110 10.1042/bj0840342 PMC1243673 13869400

[139] Li Y, Xu W, Tang Y (2010). Classification, prediction, and verification of the regioselectivity of fungal polyketide synthase product template domains. J Biol Chem.

[140] Crawford JM, Korman TP, Labonte JW, Vagstad AL, Hill EA, Kamari-Bidkorpeh O, et al. Structural basis for biosynthetic programming of fungal aromatic polyketide cyclization. Nature 2009;461:1139–43. http://www.nature.com/articles/nature08475 10.1038/nature08475 PMC2872118 19847268

[141] InterPro. Polyketide product template domain (IPR030918). Classif. protein Fam. https://www.ebi.ac.uk/interpro/entry/InterPro/IPR030918/

[142] Ahlert J, Shepard E, Lomovskaya N, Zazopoulos E, Staffa A, Bachmann BO, et al. The calicheamicin gene cluster and its iterative type I enediyne PKS. Science 2002;297:1173–6. http://science.sciencemag.org/10.1126/science.1072105 12183629

[143] Becker JE, Moore RE, Moore BS. Cloning, sequencing, and biochemical characterization of the nostocyclopeptide biosynthetic gene cluster: molecular basis for imine macrocyclization. Gene 2004;325:35–42. https://www.sciencedirect.com/science/article/pii/S0378111903009703?via%3Dihub 10.1016/j.gene.2003.09.034 14697508

[144] Ding W, Lei C, He Q, Zhang Q, Bi Y, Liu W. Insights into bacterial 6-methylsalicylic acid synthase and its engineering to orsellinic acid synthase for spirotetronate generation. Chem Biol 2010;17:495–503. https://www.sciencedirect.com/science/article/pii/S1074552110001572?via%3Dihub 10.1016/j.chembiol.2010.04.009 20534347

[145] Jia X-Y, Tian Z-H, Shao L, Qu X-D, Zhao Q-F, Tang J, et al. Genetic characterization of the chlorothricin gene cluster as a model for spirotetronate antibiotic biosynthesis. Chem Biol 2006;13:575–85. http://www.ncbi.nlm.nih.gov/pubmed/16793515 10.1016/j.chembiol.2006.03.008 16793515

[146] Gaisser S, Trefzer A, Stockert S, Kirschning A, Bechthold A. Cloning of an avilamycin biosynthetic gene cluster from Streptomyces viridochromogenes Tü57. J Bacteriol 1997;179:6271–8. http://jb.asm.org/10.1128/jb.179.20.6271-6278.1997 PMC179539 9335272

[147] Kage H, Riva E, Parascandolo JS, Kreutzer MF, Tosin M, Nett M. Chemical chain termination resolves the timing of ketoreduction in a partially reducing iterative type I polyketide synthase. Org Biomol Chem 2015;13:11414–7. http://xlink.rsc.org/?DOI=C5OB02009C 10.1039/c5ob02009c 26507693

[148] Yaegashi J, Oakley BR, Wang CCC. Recent advances in genome mining of secondary metabolite biosynthetic gene clusters and the development of heterologous expression systems in* Aspergillus nidulans*. J Ind Microbiol Biotechnol 2014;41:433–42. http://www.aspgd.org/10.1007/s10295-013-1386-z PMC4324552 24342965

[149] Chen WN, Tan KY (2013). Malonate uptake and metabolism in* Saccharomyces cerevisiae*. Appl Biochem Biotechnol.

[150] Casal M, Paiva S, Queirós O, Soares-Silva I (2008). Transport of carboxylic acids in yeasts. FEMS Microbiol Rev.

[151] Casal M, Queirós O, Talaia G, Ribas D, Paiva S. Carboxylic acids plasma membrane transporters in *Saccharomyces cerevisiae*. In: José R, Sychrová H, Kschischo M, editors. Yeast membrane transport. 2016. p. 229–51. Doi: 10.1007/978-3-319-25304-6_9 10.1007/978-3-319-25304-6_9 26721276

[152] Panagiotou G, Andersen MR, Grotkjaer T, Regueira TB, Nielsen J, Olsson L. Studies of the production of fungal polyketides in* Aspergillus nidulans* by using systems biology tools. Appl Environ Microbiol 2009;75:2212–20. http://www.fgsc.net/fgn/pall.html 10.1128/AEM.01461-08 PMC2663190 19168657

[153] Mulder KCL, Mulinari F, Franco OL, Soares MSF, Magalhães BS, Parachin NS (2015). Lovastatin production: from molecular basis to industrial process optimization. Biotechnol Adv.

[154] Zhang Y, Chen Z, Wen Q, Xiong Z, Cao X, Zheng Z, et al. An overview on the biosynthesis and metabolic regulation of monacolin K/lovastatin. Food Funct 2020;11:5738–48. http://xlink.rsc.org/?DOI=D0FO00691B 10.1039/d0fo00691b 32555902

[155] Ames BD, Nguyen C, Bruegger J, Smith P, Xu W, Ma S (2012). Crystal structure and biochemical studies of the trans-acting polyketide enoyl reductase LovC from lovastatin biosynthesis. Proc Natl Acad Sci.

[156] Barriuso J, Nguyen DT, Li JW-H, Roberts JN, MacNevin G, Chaytor JL (2011). Double oxidation of the cyclic nonaketide dihydromonacolin l to monacolin j by a single cytochrome P450 monooxygenase, LovA. J Am Chem Soc.

[157] Lee KKM, Da Silva NA, Kealey JT. Determination of the extent of phosphopantetheinylation of polyketide synthases expressed in Escherichia coli and* Saccharomyces cerevisiae*. Anal Biochem 2009;394:75–80. http://www.ncbi.nlm.nih.gov/pubmed/19595983 10.1016/j.ab.2009.07.010 19595983

[158] Wang M, Zhou H, Wirz M, Tang Y, Boddy CN. A thioesterase from an iterative fungal polyketide synthase shows macrocyclization and cross coupling activity and may play a role in controlling iterative cycling through product offloading. Biochemistry 2009;48:6288–90. http://pubs.acs.org. 10.1021/bi9009049 PMC2722786 19530704

[159] Cortes J, Haydock SF, Roberts GA, Bevitt DJ, Leadlay PF. An unusually large multifunctional polypeptide in the erythromycin-producing polyketide synthase of Saccharopolyspora erythraea. Nature 1990;348:176–8. http://www.nature.com/articles/348176a0 10.1038/348176a0 2234082

[160] Khosla C, Tang Y, Chen AY, Schnarr NA, Cane DE (2007). Structure and mechanism of the 6-deoxyerythronolide B synthase. Annu Rev Biochem.

[161] Gao X, Jiang W, Jiménez-Osés G, Choi MS, Houk KN, Tang Y, et al. An iterative, bimodular nonribosomal peptide synthetase that converts anthranilate and tryptophan into tetracyclic asperlicins. Chem Biol 2013;20:870–8. http://www.ncbi.nlm.nih.gov/pubmed/23890005 10.1016/j.chembiol.2013.04.019 PMC3728708 23890005

[162] Hühner E, Backhaus K, Kraut R, Li S-M (2018). Production of α-keto carboxylic acid dimers in yeast by overexpression of NRPS-like genes from Aspergillus terreus. Appl Microbiol Biotechnol.

[163] Tsunematsu Y, Ishiuchi K, Hotta K, Watanabe K. Yeast-based genome mining, production and mechanistic studies of the biosynthesis of fungal polyketide and peptide natural products. Nat Prod Rep 2013;30:1139–49. http://xlink.rsc.org/?DOI=c3np70037b 10.1039/c3np70037b 23824111

[164] Tsunematsu Y, Ishikawa N, Wakana D, Goda Y, Noguchi H, Moriya H, et al. Distinct mechanisms for spiro-carbon formation reveal biosynthetic pathway crosstalk. Nat Chem Biol 2013;9:818–25. https://pubmed.ncbi.nlm.nih.gov/24121553/10.1038/nchembio.1366 24121553

[165] Yu D, Xu F, Zi J, Wang S, Gage D, Zeng J, et al. Engineered production of fungal anticancer cyclooligomer depsipeptides in *Saccharomyces cerevisiae*. Metab Eng 2013;18:60–8. http://www.ncbi.nlm.nih.gov/pubmed/23608474 10.1016/j.ymben.2013.04.001 23608474

[166] Yu D, Xu F, Zhang S, Zhan J (2017). Decoding and reprogramming fungal iterative nonribosomal peptide synthetases. Nat Commun.

[167] Siewers V, San-Bento R, Nielsen J (2010). Implementation of communication-mediating domains for non-ribosomal peptide production in *Saccharomyces cerevisiae*. Biotechnol Bioeng.

[168] Xu W, Cai X, Jung ME, Tang Y. Analysis of intact and dissected fungal polyketide synthase-nonribosomal peptide synthetase in vitro and in *Saccharomyces cerevisiae*. J Am Chem Soc 2010;132:13604–7. http://pubs.acs.org. 10.1021/ja107084d PMC2950873 20828130

[169] Meijer WH, Gidijala L, Fekken S, Kiel JAKW, van den Berg MA, Lascaris R, et al. Peroxisomes are required for efficient penicillin biosynthesis in Penicillium chrysogenum. Appl Environ Microbiol 2010;76:5702–9. http://aem.asm.org/10.1128/AEM.02327-09 PMC2935065 20601503

[170] Siewers V, Chen X, Huang L, Zhang J, Nielsen J. Heterologous production of non-ribosomal peptide LLD-ACV in *Saccharomyces cerevisiae*. Metab Eng 2009;11:391–7. https://linkinghub.elsevier.com/retrieve/pii/S1096717609000640 10.1016/j.ymben.2009.08.002 19686863

[171] Müller WH, van der Krift TP, Krouwer AJJ, Wösten HA, van der Voort LH, Smaal EB (1991). Localization of the pathway of the penicillin biosynthesis in Penicillium chrysogenum. EMBO J.

[172] Müller WH, Bovenberg RAL, Groothuis MH, Kattevilder F, Smaal EB, Van der Voort LHM, et al. Involvement of microbodies in penicillin biosynthesis. Biochim Biophys Acta 1992;1116:210–3. http://www.ncbi.nlm.nih.gov/pubmed/1581347 10.1016/0304-4165(92)90118-e 1581347

[173] Martín J-F, Ullán R V., García-Estrada C. Role of peroxisomes in the biosynthesis and secretion of β-lactams and other secondary metabolites. J Ind Microbiol Biotechnol 2012;39:367–82. https://academic.oup.com/jimb/article/39/3/367/5994598 10.1007/s10295-011-1063-z 22160272

[174] Elander RP (2003). Industrial production of beta-lactam antibiotics. Appl Microbiol Biotechnol.

[175] Süssmuth R, Müller J, von Döhren H, Molnár I. Fungal cyclooligomerdepsipeptides: from classical biochemistry to combinatorial biosynthesis. Nat Prod Rep 2011;28:99–124. www.rsc.org/npr 10.1039/c001463j 20959929

[176] Xu Y, Orozco R, Wijeratne EMK, Gunatilaka AAL, Stock SP, Molnár I. Biosynthesis of the cyclooligomer depsipeptide beauvericin, a virulence factor of the entomopathogenic fungus Beauveria bassiana. Chem Biol 2008;15:898–907. http://www.ncbi.nlm.nih.gov/pubmed/18804027 10.1016/j.chembiol.2008.07.011 18804027

[177] Wang Q, Xu L (2012). Beauvericin, a bioactive compound produced by fungi: a short review. Molecules.

[178] Takahashi H, Kumagai T, Kitani K, Mori M, Matoba Y, Sugiyama M. Cloning and characterization of a Streptomyces single module type non-ribosomal peptide synthetase catalyzing a blue pigment synthesis. J Biol Chem 2007;282:9073–81. http://www.jbc.org/10.1074/jbc.M611319200 17237222

[179] Kovner A, Lawrence Berkeley National Laboratory. Blue pigment from engineered fungi could help turn the textile industry green. Lawrence Berkeley Natl. Lab. 2019. https://newscenter.lbl.gov/2019/06/21/blue-pigment-from-fungi/

[180] Sakai A, Shimizu Y, Hishinuma F (1990). Integration of heterologous genes into the chromosome of *Saccharomyces cerevisiae* using a delta sequence of yeast retrotransposon Ty. Appl Microbiol Biotechnol.

[181] Lee FWF, Da Silva NA (1997). Improved efficiency and stability of multiple cloned gene insertions at the delta sequences of *Saccharomyces cerevisiae*. Appl Microbiol Biotechnol.

[182] Banerjee D, Eng T, Lau AK, Sasaki Y, Wang B, Chen Y (2020). Genome-scale metabolic rewiring improves titers rates and yields of the non-native product indigoidine at scale. Nat Commun.

[183] Hahn M, Stachelhaus T. Selective interaction between nonribosomal peptide synthetases is facilitated by short communication-mediating domains. Proc Natl Acad Sci U S A 2004;101:15585–90. https://pubmed.ncbi.nlm.nih.gov/15498872/10.1073/pnas.0404932101 PMC524835 15498872

[184] Hahn M, Stachelhaus T (2006). Harnessing the potential of communication-mediating domains for the biocombinatorial synthesis of nonribosomal peptides. Proc Natl Acad Sci U S A.

[185] An JH, Kim YS. A gene cluster encoding malonyl-CoA decarboxylase (MatA), malonyl-CoA synthetase (MatB) and a putative dicarboxylate carrier protein (MatC) in Rhizobium trifolii—cloning, sequencing, and expression of the enzymes in *Escherichia coli*. Eur J Biochem 1998;257:395–402. https://pubmed.ncbi.nlm.nih.gov/9826185/10.1046/j.1432-1327.1998.2570395.x 9826185

[186] Gidijala L, Kiel JAKW, Douma RD, Seifar RM, van Gulik WM, Bovenberg RAL, et al. An engineered yeast efficiently secreting penicillin. PLoS One 2009;4:e8317. http://www.ncbi.nlm.nih.gov/pubmed/20016817 10.1371/journal.pone.0008317 PMC2789386 20016817

[187] Gao L, Cai M, Shen W, Xiao S, Zhou X, Zhang Y (2013). Engineered fungal polyketide biosynthesis in Pichia pastoris: a potential excellent host for polyketide production. Microb Cell Fact.

[188] Xu Q, Bai C, Liu Y, Song L, Tian L, Yan Y (2019). Modulation of acetate utilization in Komagataella phaffii by metabolic engineering of tolerance and metabolism. Biotechnol Biofuels.

[189] Xue Y, Kong C, Shen W, Bai C, Ren Y, Zhou X, et al. Methylotrophic yeast Pichia pastoris as a chassis organism for polyketide synthesis via the full citrinin biosynthetic pathway. J Biotechnol 2017;242:64–72. http://www.ncbi.nlm.nih.gov/pubmed/27913218 10.1016/j.jbiotec.2016.11.031 27913218

[190] Liu Y, Tu X, Xu Q, Bai C, Kong C, Liu Q, et al. Engineered monoculture and co-culture of methylotrophic yeast for de novo production of monacolin J and lovastatin from methanol. Metab Eng 2018;45:189–99. https://pubmed.ncbi.nlm.nih.gov/29258964/10.1016/j.ymben.2017.12.009 29258964

[191] Peña DA, Gasser B, Zanghellini J, Steiger MG, Mattanovich D. Metabolic engineering of Pichia pastoris. Metab Eng 2018;50:2–15. http://www.ncbi.nlm.nih.gov/pubmed/29704654 10.1016/j.ymben.2018.04.017 29704654

