# Supplementary material for: Saccharomyces cerevisiae as host for the recombinant production of polyketides and nonribosomal peptides
Source: Microb Cell Fact. 2021 Aug 19;20:161. doi: 10.1186/s12934-021-01650-y (PMC8374128; doi:10.1186/s12934-021-01650-y)
Supplement: Supplementary file 1 — Additional file 1: Table S1. Outline of microbial polyketides that were heterologously produced in S. cerevisiae, including information on the native producer, the relevant properties of the engineered S. cerevisiae strain, the production conditions in yeast and the titer increase compared to the native producer. Table S2. Outline of NRPS-derived secondary metabolites that were heterologously produced in S. cerevisiae, including information on the native producer, the relevant properties of the engineered S. cerevisiae strain, the production conditions in yeast and the titer increase compared to the native producer. Figure S1. Integration of polyketide and nonribosomal peptide biosynthesis into the metabolic network of S. cerevisiae. [file 12934_2021_1650_MOESM1_ESM.docx]

# Additional file 1

# *Saccharomyces cerevisiae* as host for the recombinant production of polyketides and nonribosomal peptides

Anna Tippelt and Markus Nett#

Department of Biochemical and Chemical Engineering, TU Dortmund University, Emil-Figge-Strasse 66, 44227 Dortmund, Germany

#Address correspondence to Markus Nett, markus.nett@tu-dortmund.de

**Table of contents**

Table S1. Outline of microbial polyketides that were heterologously produced in *S. cerevisiae*. SI-2

Table S2. Outline of NRPS-derived secondary metabolites that were heterologously produced in *S. cerevisiae*. SI-6

Figure S1. Integration of polyketide and nonribosomal peptide biosynthesis into the metabolic network of *S. cerevisiae*. SI-8

References SI-9

**Table S1.** Outline of microbial polyketides that were heterologously produced in *S. cerevisiae*, including information on the native producer, the relevant properties of the *S. cerevisiae* expression strain, the production conditions in yeast and the titer increase compared to the native producer. The compounds are listed in the same order as in Table 1.

| **Compound** | **Native producer** | ***S. cerevisae* expression strain** | **Relevant properties of the expression strain yielding the highest titer** | **Production conditions (medium,^a^ fermentation mode)** | **Titer increase compared to native producer** | **Literature** |
| --- | --- | --- | --- | --- | --- | --- |
| **TAN-1612 and derivatives** | *Aspergillus niger* | BJ5464-NpgA | *MATα ura3-52 his3-∆200 leu2-∆1 trp1 pep4::HIS3 prb1∆1.6R can1 GAL*  pYR291 carrying *2µ ori*, *pMB1 ori, f1 ori, Amp^R^, URA3,* ADH2p-*adaA*-ADH2t  pYR342 carrying *2µ ori*, *pMB1 ori, f1 ori, Amp^R^,* *TRP1*, ADH2p-*adaB*-ADH2t, ADH2p-*adaC*-ADH2t, and ADH2p-*adaD*-ADH2t  (AdaA, NRPKS; AdaB, thioesterase; AdaC, flavin-dependent oxidoreductase; AdaD, methyltransferase) | YPD  batch | 3.4x | [1] |
| **emodin** | *Shiraia* sp. | BJ5464-NpgA | *MATα ura3-52 his3-Δ200 leu2-Δ1 trp1 pep4::HIS3 prb1 Δ1.6R can1 GAL*  pSUN39 carrying *2µ ori, pBR322 ori, Amp^R^, URA3,* ADH2p*-emoA*  pSUN143 carrying *2µ ori*, *pBR322 ori*, *Amp^R^*, *TRP1*, ADH2p-*acte1*  pSUN164 carrying *2µ ori, f1 ori, Amp^R^, leu2,* ADH2p-*ACC1^S1157A^*  pSUN207 carrying *2µ ori, f1 ori, Amp^R^, leu2,* ADH2p-*tpcK*  (EmoA, NRPKS; Acte1, thioesterase; ACC1, acetyl-CoA carboxylase; TpcK, decarboxylase) | YPD (+ pyruvate)  batch | not reported | [2] |
| **endocrocin** | *Shiraia* sp. | BJ5464-NpgA | *MATα ura3-52 his3-Δ200 leu2-Δ1 trp1 pep4::HIS3 prb1 Δ1.6R can1 GAL*  pSUN39 carrying *2µ ori, pBR322 ori, Amp^R^, URA3,* ADH2p*-emoA*  pSUN143 carrying *2µ ori*, *pBR322 ori*, *Amp^R^*, *TRP1*, ADH2p-*acte1*  pSUN164 carrying *2µ ori, f1 ori, Amp^R^, leu2,* ADH2p-*ACC1^S1157A^*  (EmoA, NRPKS; Acte1, thioesterase; ACC1, acetyl-CoA carboxylase) | SC, YPD (+ pyruvate)  batch | not reported | [2] |
| **DMAC,**  **dihydro-kalafungin** | *Aloe arborescens*, *Streptomyces coelicolor* | CEN.PK2–1C | *MATa his3D1 leu2-3_112 ura3-52 trp1-289 MAL2-8c SUC2*  X-3: TDH3p-*actIII*-tADH1 and TEF1p-a*ctIV*-tCYC1  XI-2: TDH3p-*actVI-1*-tADH1 and TEF1p-*actVI-3*-tCYC1  XII-2: TDH3p-*actVII*-tADH1 and TEF1p-*actVI-A*-tCYC1  X-2: TDH3p-*actVI-4*-tADH1 and TEF1p-*actVA-6*-tCYC1  XI-5: TEF1p-*actVI-2*-tCYC1  XII-4: TDH3p-*actVA-5*-tADH1 and TEF1p-*actVB*-tCYC1  pTAJAK-205 (pESC-derived) carrying *2µ ori, f1 ori, Amp^R^, URA3,* and TDH3p-*aaOKS*-tCYC1  (AaOKS, octaketide synthase from *Aloe arborescens*; ActIII-VII, actinorhodin biosynthesis enzymes from *Streptomyces coelicolor*) | SC, YPD  batch | not reported | [3] |
| **(*nor*)-rubrofusarin** | *Fusarium graminearum* | BY4742-derived | *MATα, trp1Δ, lys2Δ0, leu2Δ, his3Δ1, ura3Δ0, arg4::loxP*  pRS413 carrying *CEN/ARS, f1 ori, ColE1 ori, Amp^R^, HIS3,* PYK1p*-npgA-*TEF1t  pRS416 carrying *CEN/ARS, f1 ori, ColE1 ori, Amp^R^, URA3,* CUP1p*-pks12-*ADH1t  pRS414 carrying *CEN/ARS, f1 ori, ColE1 ori, Amp^R^, TRP1,* TEF1p*-aurZ-*ENO2t  pRS415 carrying *CEN/ARS, f1 ori, ColE1 ori, Amp^R^, LEU2,* GPD1p*-aurJ-*CYC1t  (NpgA, PPTase; AurZ, dehydratase; AurJ, *O*-methyltransferase) | SC  batch | titer is reduced | [4, 5] |
| **brefeldin A precursors** | *Eupenicillium brefeldianum* | BJ5464-NpgA | *MATα ura3-52 his3-∆200 leu2-∆1 trp1 pep4::HIS3 prb1∆1.6R can1 GAL*  pAZ94 carrying *2µ ori, pBR322 ori, Amp^R^, URA3,* ADH2p*-brefPKS*  pAZ112 carrying *2µ ori, TRP3,* ADH2p*-brefTH*  (BrefPKS, HRPKS; BrefTH, thioesterase) | YPD  batch | not reported | [6] |
| **monocillin II and pochonin D** | *Pochonia chlamydosporia* | BJ5464-NpgA | *MATα ura3-52 his3-∆200 leu2-∆1 trp1 pep4::HIS3 prb1∆1.6R can1 GAL*  pKJ61 carrying *2µ ori, pBR322 ori, Amp^R^, URA3,* ADH2p*-rdc5*  pKJ91 carrying *2µ ori, pBR322 ori, Amp^R^, TRP1,* ADH2p*-rdc1*  (Rdc5, HRPKS; Rdc1, NRPKS) | YPD  batch | not reported | [7] |
| **7',8'-dehydro-zearalenol** | *Hypomyces subiculosus* | BJ5464-NpgA | *MATα ura3-52 his3-∆200 leu2-∆1 trp1 pep4::HIS3 prb1∆1.6R can1 GAL* pKOS518-118A carrying *2µ ori, pBR322 ori, f1 ori ,Amp^R^, LEU2,* ADH2p*-hpm8-*ADH2t  pKOS518-120A carrying *2µ ori, pBR322 ori, f1 ori, Amp^R^, TRP1,* ADH2p*-hpm3-*ADH2t  (Hpm8, HRPKS; Hpm3, NRPKS;; Hpm5) | YPD  batch | not reported | [8-10] |
| **10,11-dehydro-curvularin** | *Alternaria cinerariae* | BJ5464-NpgA | *MATα ura3-52 his3-∆200 leu2-∆1 trp1 pep4::HIS3 prb1∆1.6R can1 GAL*  pKOS518–120A carrying *2µ ori*,  *pBR322 ori, f1 ori, Amp^R^, TRP1,*, ADH2p-*dhc3*-ADH2t  pXK30 carrying *2µ ori*,  *pBR322 ori, f1 ori, Amp^R^, URA3*, ADH2p-*dhc5*-ADH2t  (Dhc3, HRPKS; Dhc5, NRPKS) | YPD  batch | titer is reduced | [11, 12] |
| ***trans*-resorcylide,**  **zearalane, lasicicol, 10,11-dehydro-curvularin, …** | varied | BJ5464-NpgA | *MATα ura3-52 his3-∆200 leu2-∆1 trp1 pep4::HIS3 prb1∆1.6R can1 GAL*  YEpAt-CURS1 carrying *2µ ori, Amp^R^, TRP1*, ADH2p-*hrpks*  YEpAtCURS2 carrying *2µ ori, Amp^R^, URA3*, ADH2p-*nrpks*  (HRPKS: AtCurS1, CcRadS1, AzResS1, or LtLasS1; NRPKS: AtCurS2, CcRadS2, AzResS2, or LtLasS2) | YPD  batch | 1.8x (for 10,11-dehydro-curvularin) | [13, 14] |
| **monocillin II, 10,11-dehydro-curvularin, lasicicol, lasilarin, radilarin, radiplodin** | -  (combinatorial) | BJ5464-NpgA | see previous entry;  combinatorial biosynthesis through coexpression of HRPKSs and NRPKSs from different pathways | YPD  batch | not applicable | [14] |
| **bikaverin** | *Fusarium fujikuroi* | BY4742 | *MATα ura3∆0 leu2∆0 lys2∆0 his3∆1*  pZM-y037 carrying *CEN/ARS, URA3, pBR322 ori, f1 ori ,Amp^R^,* Gal1p-*bik2-bik3*-CYC1t, Gal1p-*bik1*-ACS1t*,* PGK1p*-bik6*-ZEO1t, ENO2p-*ppt1-*ADH2t, and TEF2p-*npgA*-HXT7t  (Bik1, NRPKS; Bik2, monooxygenase; Bik3, O-methyltransferase; Bik6, permease; NpgA, PPTase; Ppt1, PPTase from *F. fujikuroi*) | YPD  batch | not reported | [15] |
| **mellein** | *Parastagonospora nodorum* | BJ5464-NpgA | *MATα ura3-52 his3-∆200 leu2-∆1 trp1 pep4::HIS3 prb1∆1.6R can1 GAL*  YEplac carrying *2µ ori*, *pBR322 ori, Amp^R^, URA3*, ADH2p-*SN477*  (SN477, PRPKS) | YPD  batch | not reported | [16] |
| **de-*O*-methyl-diaporthin** | *Chaetomium globosum* | SCKW5  (BY4741-derived) | *MATa his3-∆1 leu2-∆0 met15- ∆0 ura3-∆0 ∆FCY1::matB-npgA-kanMX*  pKW14051 carrying *2µ ori*, *pBR322 ori, Amp^R^* , *HIS3*, TEF1p-*CHGG_00542**-CYC1t  (MatB, malonyl-CoA synthetase; NpgA, PPTase; KanMX, G418 resistance marker; CHGG_00542*, S2010A mutant of NRPKS) | YPD  batch | not reported | [17] |
| **chaetoviridin A and cazaldehyde precursor** | *Chaetomium globosum* | BJ5464-NpgA | *MATα ura3-52 his3-∆200 leu2-∆1 trp1 pep4::HIS3 prb1∆1.6R can1 GAL*  pJWT-41 carrying *2µ ori*,  *pMB1 ori, f1 ori, Amp^R^, TRP1,* ADH2p*-cazF*-ADH2t  pJWT-37 carrying *2µ ori*,  *pMB1 ori, f1 ori, Amp^R^, URA3,* ADH2p*-cazM*-ADH2t  ( CazF, HRPKS CazM, NRPKS) | YPD  batch | not reported | [18] |
| **triketide lactone** | -  (combinatorial) | K283-68-1 (BJ5464- derived) | *MATα ura3-52 his3-∆200 leu2-∆1 trp1 pep4::HIS3 prb1∆1.6R can1 GAL*  *sfp* and *prpE* were integrated into the yeast chromosomal δ-sequences  pKOS266-46-40 carrying *2µ ori*, *pMB1 ori, f1 ori, Amp^R^, URA3*, ADH2p-*pccB*, and ADH2p-*accA*  pKOS253-179-28 carrying *2µ ori*, *pMB1 ori, f1 ori, Amp^R^, TRP1,* and ADH2p*-DEBS2-TE*  pKOS283-31-19 carrying *CEN/ARS ori, f1 ori, pMB1 ori, Amp^R^, HIS3,* *tRNA genes* (*E4, R2, L5, Q2, P2*), ADH2p-*birA*  (Sfp, PPTase; PrpE, propionyl-CoA synthetase; PccB, transcarboxylase subunit of propionyl-CoA carboxylase (PCC); AccA, biotin carboxylase subunit of PCC; DEBS2-TE, module 2 of 6-deoxyerythronolide B synthase fused to thioesterase domain; BirA, bifuctional biotin-acetyl-CoA-carboxylase/ligase) | YPD (+ propyl-diketide *N*-acetylcysteamine thioester)  batch | not applicable | [19-21] |
| **3-ethylphenol, 3-propylphenol** | -  (combinatorial) | JHY218 and JHY212 (CEN.PK2-1C-derived) | 3-ethylphenol production: *MATa leu2-3,112 ura3-52 trp1-289 his3-Δ1 MAL2–8c SUC2 sfa1p-sfa1Δ::*TDH3p*-^Stopt^prpE-*SFA1t, *ura3::*PGK1p*-^Ppopt^msas-*CYC1t, HXT7p*-^Anopt^npgA-*FBA1t, FBA1p*-^Acopt^patG-*ADH1t  3-propylphenol production: *MATa leu2-3,112 ura3-52 trp1-289 his3-Δ1 MAL2–8c SUC2 pox1Δ ura3::*PGK1p*-^Ppopt^msas-*CYC1t*,* HXT7p*-^1–392-^ ^Anopt^npgA-*FBA1t*,*FBA1p*-^Acopt^patG-*ADH1t *leu2::*PGK1p*-^Sc^ERG10-*VMA16t*,* CCW12p*-^Caopt^hbd-*IDPt*,* ENO2p*-^Caopt^crt-*PGK1t*,* TDH3p*-^Tdopt^tert-*ADH1t*,*TEFp*-natMX-*TEFt  (^Stopt^PrpE, codon optimized propionyl-CoA synthetase; ^Ppopt^MSAS, codon optimized 6-methylsalicylic acid synthase*;* ^Anopt^NpgA, codon optimized PPTase; ^Acopt^PatG, codon optimized 6-MSA decarboxylase; ^Sc^ERG10*,* thiolase*;* ^Caopt^Hbd, codon optimized hydroxybutyryl-CoA dehydrogenase; ^Caopt^Crt, codon optimized crotonase; ^Tdopt^Ter, codon optimized *trans*-2-enoyl-CoA reductase; NatMX, nourseothricin resistance marker) | YPD  batch | not applicable | [22] |
| ***m*-cresol** | -  (combinatorial) | CEN.PK2–1C | *MATa leu2-3112 ura3-52 trp1-289 his3-Δ1 MAL2–8c SUC2 ura3*::PGK1p-*^Ppopt^msas*-CYC1t_HXT7p^-^*^1- -392^ -^Anopt^npgA*-FBA1t_FBA1p-*^Acopt^patG*-ADH1t  pJHV53 carrying *2µ ori*, *f1 ori*, pMB1 *ori, Amp^R^, kanMX*, PGK1p-*^Ppopt^msas*-CYC1t, HXT7p-*^Anopt^npgA*-FBA1t, and FBA1p-*^Acopt^patG*-ADH1t  (^Anopt^NpgA, codon optimized PPTase*;* ^Ppopt^MSAS*,* codon optimized 6-methyl-salicylic acid synthase;  ^Acopt^PatG, codon optimized 6-MSA decarboxylase; KanMX, G418 resistance marker) | YPD  batch | not applicable | [23] |
| **5-methyl-**  **orsellinic acid** | *Aspergillus terreus* | HZ848 | *MATα ade2-1 Δura3 his3-11, 15 trp1-1 leu2-3 112 can1-100*  pRS416-derived plasmid carrying *CEN6_ARS4, ColE1 ori, f1 ori*, *Amp^R^*, *URA3*, and GPM1p-*npgA*-Gpm1t  pRS414-derived plasmid carrying *CEN6_ARS4, ColE1 ori, f1 ori*, *Amp^R^*, *TRP1*, and TEF1p-*ATEG_03629*-HXT7t  (NpgA, PPTase; ATEG_03629, NRPKS) | SC  batch | not reported | [24] |
| **6-methyl-**  **orsellinic acid** | *Chaetomium globosum* | SCKW5  (BY4741-derived) | *MATa his3-∆1 leu2-∆0 met15- ∆0 ura3-∆0 ∆FCY1::matB-npgA-kanMX*  pKW14051 carrying *2µ ori*, *pBR322 ori, Amp^R^* , *HIS3*, TEF1p-*CHGG_10128*-CYC1t  (MatB, malonyl-CoA synthetase; NpgA, PPTase; KanMX, G418 resistance marker; CHGG_10128, NRPKS) | YPD  batch | not reported | [17] |
| **6-methyl-**  **salicylic acid** | *Penicillium patulum* | CEN.PK2–1C | *MATa leu2-3112 ura3-52 trp1-289 his3-Δ1 MAL2–8c SUC2 ura3*::PGK1p-*^Ppopt^msas*-CYC1t_HXT7p^-^*^1- -392^ -^Anopt^npgA*-FBA1t  pJHV49 carrying *2µ ori*, *f1 ori*, pMB1 *ori, Amp^R^, kanMX*, PGK1-*^Ppopt^msas*-CYC1t, and HXT7p-*^Anopt^npgA*-FBA1t  (^Anopt^NpgA, codon optimized PPTase*;* ^Ppopt^MSAS*,* codon optimized 6-methyl-salicylic acid synthase; KanMX, G418 resistance marker) | YPD  batch | 20,000x | [23, 25] |
| **orsellinic acid** | *Coprinopsis cinerea* | SCKW5  (BY4741-derived) | *MATa his3-∆1 leu2-∆0 met15- ∆0 ura3-∆0 ∆FCY1::matB-npgA-kanMX*  pKW14051 carrying *2µ ori*, *pBR322 ori, Amp^R^* , *HIS3*, TEF1p-*CC1GG_05377*-CYC1t  (MatB, malonyl-CoA synthetase; NpgA, PPTase; KanMX, G418 resistance marker; CC1G_05377, NRPKS) | YPD  batch | titer is reduced | [17, 26] |
| **monacolin L and J acid** | *Aspergillus terreus* | BJ5464-NpgA | *MATα ura3-52 his3-∆200 leu2-∆1 trp1 pep4::HIS3 prb1∆1.6R can1 GAL* *ura3Δ::*ADH2p*-cpr-*ADH2t and *pyc2∆::*ADH2p*-lovA-*ADH2t  pLovB carrying *2µ ori, Amp^R^*, *URA3,* ADH2p-*lovB-*6xHis*-*ADH2t  pCB21 carrying *2µ ori, Amp^R^, TRP1,* ADH2p*-*6xHis*-mlcG-*ADH2t*,* and ADH2p*-lovG-*6xHis-ADH2t  pCB19 carrying *2µ ori, Amp^R^, LEU2,* ADH2p*-lovA-*6xHis-ADH2t*,* and ADH2p-6xHis*-lovD9*-ADH2t  (Cpr, cytochrome P450 reductase; LovA, cytochrome P450 monooxygenase; LovB, highly reducing PKS; MlcG, LovC homolgous enoyl reductase; LovG, thioesterase; LovD9, engineered thioesterase-like acyltransferase) | YPD  batch | not reported | [27] |
| **simvastatin**  (= semisynthetic derivative of lovastatin) | - | BJ5464-NpgA | see entry for monacolin L and J acid | YPD  batch; addition of semi-synthetic acyl donor after cell lysis | not applicable | [27] |

^a^Media abbreviations: SC, synthetic complete medium minus the appropriate amino acids or nucleotide for selective pressure with either glucose or galactose as the carbon source; YPD, yeast extract-peptone-dextrose

**Table S2.** Outline of NRPS-derived secondary metabolites that were heterologously produced in S. cerevisiae, including information on the native producer, the relevant properties of the S. cerevisiae expression strain, the production conditions in yeast and the titer increase compared to the native producer. The compounds are listed in the same order as in Table 2.

| **Compound** | **Native producer** | ***S. cerevisae* expression strain** | **Relevant properties of the expression strain yielding the highest titer** | **Production conditions (medium,^a^ fermenta-tion mode)** | **Titer increase compared to native producer** | **Literature** |
| --- | --- | --- | --- | --- | --- | --- |
| **benzylpenicillin** | *Penicillium chrysogenum* | Sc.P1  (BY4741-derived) | *MATa his3-Δ1 leu2-Δ0 met15-Δ0 ura3-Δ0*  *TRP1* gene was replaced with pGAL10-*pcbAB* and GAL1p-*npg*  (PcbAB, ACV synthetase; NpgA, PPTase)  pAA056 carrying *2µ ori*, *ColE1 ori*,  *Cam^R^*, *HIS3*, GAL1p-*pcbC*, CCW12p-*penDE*-PTS1, and TDH3p-*pclA*-PTS1  (PcbC, isopenicillin N synthase; PenDE, isopenicillin N-acyltransferase; PclA, phenylacetyl-CoA ligase; PTS1, peroxisome targeting sequence tag) | SC  batch | titer is reduced (cf. main manuscript text) | [28] |
| **asperlicin C/D** | *Aspergillus alliaceus* | BJ5464-NpgA | *MATα ura3-52 his3-Δ200 leu2-Δ1 trp1 pep4::HIS3 prb1 Δ1.6R can1 GAL*  plasmid carrying *2µ ori*, *pBR322 ori*, *Amp^R^*, *URA3*, and *aspA*  (AspA, asperlicin synthetase) | YPD  batch | not reported; *in vitro* biosynthesis after purification of recombinant AspA | [29] |
| **benzo-diazepinedione** | *Neosartorya fischeri* | BJ5464-NpgA | *MATα ura3-52 his3-Δ200 leu2-Δ1 trp1 pep4::HIS3 prb1 Δ1.6R can1 GAL*  plasmid carrying *2µ ori*, *pBR322 ori*, *Amp^R^*, *URA3*, and ADH2p-*anaPS*  (AnaPS, acetylaszonalenin synthetase) | YPD  batch | not reported | [30] |
| **atromentin** | *Aspergillus terreus* | BJ5464-NpgA | *MATα ura3-52 his3-Δ200 leu2-Δ1 trp1 pep4::HIS3 prb1 Δ1.6R can1 GAL*  pEH55 (pESC-derived) carrying *2µ ori*,  *Amp^R^*, *URA3*, and *atrA_At_*  (AtrA_At_, atromentin synthetase from *A. terreus*) | SC  batch | not reported | [31] |
| **tryprostatin B** | *Aspergillus fumigatus* | SCKW5 | *MATa his3-∆1 leu2-∆0 met15- ∆0 ura3-∆0 ∆FCY1::matB-npgA-kanMX*  pKW5011 carrying *2µ ori*, *pBR322 ori*, *Amp^R^*, *HIS3*, *leu2d*, and GAL1p-*ftmB*; pKW5054 carrying *2µ ori*, *pBR322 ori*, *Amp^R^*, *URA3*, *leu2d*, GAL1p-*ftmA*, HXT7p-*ftmD,* and TEF1p-*ftmC*; pKW5072 carrying *2µ ori*, *pBR322 ori*, *Amp^R^*, *MET15*, *leu2d*, and GAL1p-*NCP1*  (MatB, malonyl-CoA synthetase; NpgA, PPTase; KanMX, G418 resistance marker; FtmA, NRPS; FtmB, prenyltransferase; FtmC, cytochrome P450; FtmD, methyltransferase; NCP1, cytochrome P450 reductase) | YPD  batch | 93.0x | [32] |
| **bassianolide** | *Beauveria bassiana* | BJ5464-NpgA | *MATα ura3-52 his3-Δ200 leu2-Δ1 trp1 pep4::HIS3 prb1 Δ1.6R can1 GAL*  pDY42 carrying *2µ ori*, *pBR322 ori*, *Amp^R^*, *URA3*, and ADH2p-*bbBSLS*  (BbBSLS, bassianolide synthetase) | YPD  batch | 1.0x (with pDY42) | [33] |
| **beauvericin** | *Beauveria bassiana* | BJ5464-NpgA | *MATα ura3-52 his3-Δ200 leu2-Δ1 trp1 pep4::HIS3 prb1 Δ1.6R can1 GAL*  pDY37 carrying *2µ ori*, *pBR322 ori*, *Amp^R^* , *URA3*, and ADH2p-*bbBEAS*  pDY48 carrying *2µ ori*, *pBR322 ori*, *Amp^R^* , *TRP1*, and ADH2p-*kivr*  (BbBEAS, beauvericin synthetase; KIVR, ketoisovalerate reductase) | YPD  batch | 1.0x (only pDY37)  2.6x (+ pDY48)  4.5x (+ pDY48 + feeding of l-Val) | [33] |
| **phenguignardic acid** | *Aspergillus terreus* | BJ5464-NpgA | *MATα ura3-52 his3-Δ200 leu2-Δ1 trp1 pep4::HIS3 prb1 Δ1.6R can1 GAL*  pEH27 (pESC-derived) carrying *2µ ori*,  *Amp^R^*, *URA3*, and *pgnA*  (PgnA, phenguignardic acid synthetase) | SC  batch | not reported | [31] |
| **D-Phe–L-Leu dipeptide** | -  (combinatorial) | CEN.PK113-11C | *MATa MAL2-8^c^ SUC2 ura3-52 his3-Δ1*  pESC-derived plasmid carrying *2µ ori*,  *Amp^R^*, *URA3*, *sfp* and *tycA*  pESC-derived plasmid carrying *2µ ori*,  *Amp^R^*, *HIS3*, and *srfAC*  (Sfp, PPTase; TycA, tyrocidine synthetase; SrfAC, surfactin synthetase) | SG  batch | not applicable | [34] |
| **aspulvinone E** | *Aspergillus terreus* | BJ5464-NpgA | *MATα ura3-52 his3-Δ200 leu2-Δ1 trp1 pep4::HIS3 prb1 Δ1.6R can1 GAL*  pEH10 (pESC-derived) carrying *2µ ori*,  *Amp^R^*, *URA3*, and a*ApvA*  (ApvA, aspulvinone synthetase) | SC  batch | not reported | [31] |
| **butyrolactone IIa** | *Aspergillus terreus* | BJ5464-NpgA | *MATα ura3-52 his3-Δ200 leu2-Δ1 trp1 pep4::HIS3 prb1 Δ1.6R can1 GAL*  pEH29 (pESC-derived) carrying *2µ ori*,  *Amp^R^*, *URA3*, and *btyA*  (BtyA, butyrolactone synthetase) | SC  batch | not reported | [31] |
| **2,4-dihydroxy-5,6-dimethyl-benzaldehyde** | *Aspergillus terreus* | HZ848 | *MATα ade2-1 Δura3 his3-11, 15 trp1-1 leu2-3 112 can1-100*  pRS416-derived plasmid carrying *CEN6_ARS4, ColE1 ori, f1 ori*, *Amp^R^*, *URA3*, and GPM1p-*npgA*-Gpm1t  pRS414-derived plasmid carrying *CEN6_ARS4, ColE1 ori, f1 ori*, *Amp^R^*, *TRP1*, TEF1p-*ATEG_03629*-HXT7t, and GPDp-*ATEG_03630*-PYK1t  (NpgA, PPTase; ATEG_03629, NRPKS; ATEG_03630, NRPS-like protein) | SC  batch | not reported | [24] |
| **indigoidine** | *Streptomyces lavendulae* | BJ5465 | *MATa ura3-52 his3-Δ200 leu2-Δ1 trp1 pep4::HIS3 prb1 Δ1.6R can1 GAL*  *sfp* was integrated into the yeast chromosomal δ-sequences  TDH3p-*bpsA*-ADH1t was integrated into locus ARS1014a  (Sfp, PPTase; BpsA, blue pigment synthetase) | YPD  batch or fed-batch | 13.3x (batch with galactose)  26.7x (batch with glucose)  65.3x (fed-batch with glucose and carbon depletion) | [35, 36] |
| **preaspyridone** | *Aspergillus nidulans* | BJ5464-NpgA | *MATα ura3-52 his3-Δ200 leu2-Δ1 trp1 pep4::HIS3 prb1 Δ1.6R can1 GAL*  pXW58 carrying *2µ ori*, *URA3*, and ADH2p-*apdA*  pXW51 carrying *2µ ori*, TRP1, and ADH2p-*apdC*  (ApdA, aspyridone synthetase; ApdC, enoylreductase) | YPD  batch | not reported | [37] |
| **(7-hydroxy)-fumiquinazoline F** | *Penicillium aethiopicum* | BJ5464-NpgA | *MATα ura3-52 his3-Δ200 leu2-Δ1 trp1 pep4::HIS3 prb1 Δ1.6R can1 GAL*  plasmid carrying *2µ ori*, *pBR322 ori*, *Amp^R^*, *URA3*, and ADH2p-*TqaA*  (TqaA, tryptoquialanine synthetase) | YPD  batch | not reported | [30] |

^a^Media abbreviations: SC, synthetic complete medium minus the appropriate amino acids or nucleotide for selective pressure with either glucose or galactose as the carbon source; YPD, yeast extract-peptone-dextrose; SG, synthetic galactose minimal medium

**
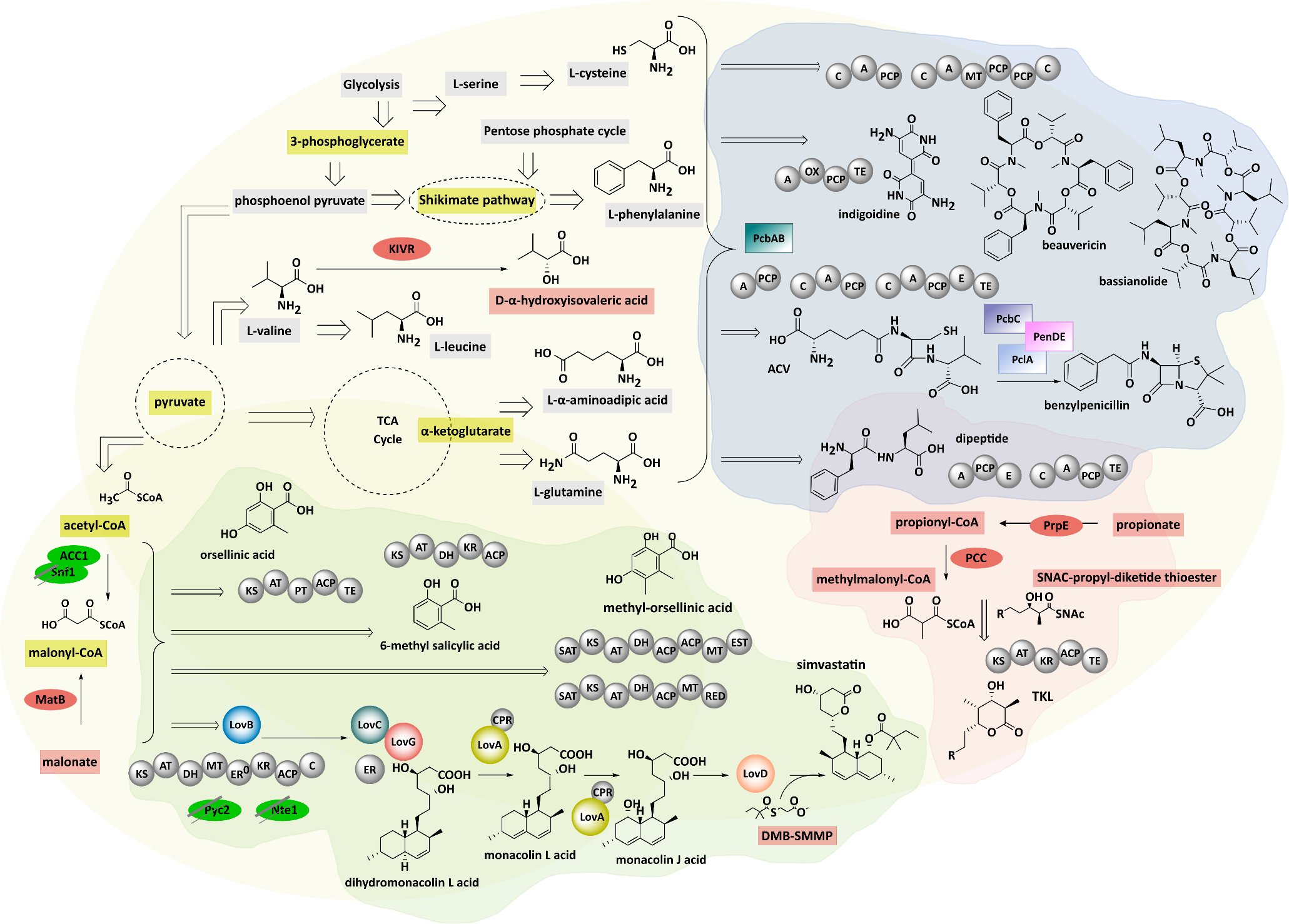
**

**Figure S1.** Integration of polyketide (green background) and nonribosomal peptide biosynthesis (blue background) into the metabolic network of S. cerevisiae. Metabolites that do not naturally occur in yeast are highlighted with a red background coloration. Red ellipses indicate heterologously expressed enzymes for precursor supply. Green ellipses highlight intrinsic pathway enzymes, which have been engineered in order to improve precursor pools. Inactivated pathway are crossed out. Pathway intermediates highlighted in yellow refer to metabolic branching points that are relevant for the reconstituted compounds.

**References**

1. Li Y, Chooi Y-H, Sheng Y, Valentine JS, Tang Y. Comparative characterization of fungal anthracenone and naphthacenedione biosynthetic pathways reveals an α-hydroxylation-dependent Claisen-like cyclization catalyzed by a dimanganese thioesterase. J Am Chem Soc 2011;133:15773–15785.

2. Sun L. Investigation and Engineering of Polyketide Biosynthetic Pathways. All Grad. Theses Diss. 6903. Utah State University; 2017. Available from: https://digitalcommons.usu.edu/etd/6903

3. Jakočiūnas T, Klitgaard AK, Kontou EE, Nielsen JB, Thomsen E, Romero-Suarez D, Blin K, Petzold CJ, Gin JW, Tong Y, Gotfredsen CH, Charusanti P, Frandsen RJN, Weber T, Lee, SY, Jensen MK, Keasling JD. Programmable polyketide biosynthesis platform for production of aromatic compounds in yeast. Synth Syst Biotechnol 2020;5:11–18.

4. Ashley JN, Hobbs BC, Raistrick H. Studies in the biochemistry of micro-organisms: The crystalline colouring matters of *Fusarium culmorum* (W. G. Smith) Sacc. and related forms. Biochem J 1937;31:385–397.

5. Rugbjerg P, Naesby M, Mortensen UH, Frandsen RJN. Reconstruction of the biosynthetic pathway for the core fungal polyketide scaffold rubrofusarin in *Saccharomyces cerevisiae*. Microb Cell Fact 2013;12:31.

6. Zabala AO, Chooi Y-H, Choi MS, Lin H-C, Tang Y. Fungal polyketide synthase product chain-length control by partnering thiohydrolase. ACS Chem Biol 2014;9:1576–1586.

7. Zhou H, Qiao K, Gao Z, Vederas JC, Tang Y. Insights into radicicol biosynthesis via heterologous synthesis of intermediates and analogs. J Biol Chem 2010;285:41412–41421.

8. Kealey JT, Liu L, Santi D V, Betlach MC, Barr PJ. Production of a polyketide natural product in nonpolyketide-producing prokaryotic and eukaryotic hosts. Proc Natl Acad Sci U S A 1998;95:505–509.

9. Reeves CD, Hu Z, Reid R, Kealey JT. Genes for the Biosynthesis of the fungal polyketides hypothemycin from *Hypomyces subiculosus* and radicicol from *Pochonia chlamydosporia*. Appl Environ Microbiol 2008;74:5121–5129.

10. Zhou H, Qiao K, Gao Z, Meehan MJ, Li JWH, Zhao X, Dorrestein P, Vederas JC, Tang Y. Enzymatic synthesis of resorcylic acid lactones by cooperation of fungal iterative polyketide synthases involved in hypothemycin biosynthesis. J Am Chem Soc 2010;132:4530–4531.

11. Robeson DJ, Strobel GA, Strange RN. The Identification of a major phytotoxic component from *Alternaria macrospora* as αβ-dehydrocurvularin. J Nat Prod 1985;48:139–141.

12. Cochrane RVK, Gao Z, Lambkin GR, Xu W, Winter JM, Marcus SL, Tang Y, Vederas JC. Comparison of 10,11-dehydrocurvularin polyketide synthases from *Alternaria cinerariae* and *Aspergillus terreus* highlights key structural motifs. Chembiochem 2015;16:2479–2483.

13. Zhan J, Wijeratne EMK, Seliga CJ, Zhang J, Pierson EE, Pierson LS, Vanetten HD, Gunatilaka AAL. A new anthraquinone and cytotoxic curvularins of a *Penicillium* sp. from the rhizosphere of *Fallugia paradoxa* of the Sonoran desert. J Antibiot (Tokyo) 2004;57:341–344.

14. Xu Y, Zhou T, Zhang S, Espinosa-Artiles P, Wang L, Zhang W, Lin M, Gunatilaka AAL, Zhan J, Molnár I. Diversity-oriented combinatorial biosynthesis of benzenediol lactone scaffolds by subunit shuffling of fungal polyketide synthases. Proc Natl Acad Sci U S A 2014;111:12354–12359.

15. Zhao M, Zhao Y, Yao M, Iqbal H, Hu Q, Liu H, Qiao B, Li C, Skovbjerg CAS, Nielsen JC, Nielsen J, Frandsen RJN, Yuan Y, Boeke JD. Pathway engineering in yeast for synthesizing the complex polyketide bikaverin. Nat Commun 2020;11:6197.

16. Chooi Y-H, Krill C, Barrow RA, Chen S, Trengove R, Oliver RP, Solomon PS. An in planta-expressed polyketide synthase produces (*R*)-mellein in the wheat pathogen *Parastagonospora nodorum*. Appl Environ Microbiol 2015;81:177–186.

17. Ishiuchi K, Nakazawa T, Ookuma T, Sugimoto S, Sato M, Tsunematsu Y, Ishikawa N, Noguchi H, Hotta K, Moriya H, Watanabe K. Establishing a new methodology for genome mining and biosynthesis of polyketides and peptides through yeast molecular genetics. ChemBioChem 2012;13:846–854.

18. Winter JM, Cascio D, Dietrich D, Sato M, Watanabe K, Sawaya MR, Vederas JC, Tang Y. Biochemical and structural basis for controlling chemical modularity in fungal polyketide biosynthesis. J Am Chem Soc 2015;137:9885–9893.

19. Hani J, Feldmann H. tRNA genes and retroelements in the yeast genome. Nucleic Acids Res 1998;26:689–696.

20. Barker DF, Campbell AM. The *birA* gene of *Escherichia coli* encodes a biotin holoenzyme synthetase. J Mol Biol 1981;146:451–467.

21. Mutka SC, Bondi SM, Carney JR, Da Silva NA, Kealey JT. Metabolic pathway engineering for complex polyketide biosynthesis in *Saccharomyces cerevisiae*. FEMS Yeast Res 2006;6:40–47.

22. Hitschler J, Grininger M, Boles E. Substrate promiscuity of polyketide synthase enables production of tsetse fly attractants 3-ethylphenol and 3-propylphenol by engineering precursor supply in yeast. Sci Rep 2020;10:9962.

23. Hitschler J, Boles E. De novo production of aromatic *m*-cresol in *Saccharomyces cerevisiae* mediated by heterologous polyketide synthases combined with a 6-methylsalicylic acid decarboxylase. Metab Eng Commun 2019;9:e00093.

24. Wang M, Beissner M, Zhao H. Aryl-aldehyde formation in fungal polyketides: discovery and characterization of a distinct biosynthetic mechanism. Chem Biol 2014;21:257–263.

25. Wattanachaisaereekul S, Lantz AE, Nielsen ML, Andrésson ÓS, Nielsen J. Optimization of heterologous production of the polyketide 6-MSA in *Saccharomyces cerevisiae*. Biotechnol Bioeng 2007;97:893–900.

26. Sanchez JF, Chiang Y-M, Szewczyk E, Davidson AD, Ahuja M, Elizabeth Oakley C, Wang CCC. Molecular genetic analysis of the orsellinic acid/F9775 gene cluster of *Aspergillus nidulans*. Mol Biosyst 2010;6:587–593.

27. Bond CM, Tang Y. Engineering *Saccharomyces cerevisiae* for production of simvastatin. Metab Eng 2019;51:1–8.

28. Awan AR, Blount BA, Bell DJ, Shaw WM, Ho JCH, McKiernan RM, Ellis T. Biosynthesis of the antibiotic nonribosomal peptide penicillin in baker’s yeast. Nat Commun 2017;8:15202.

29. Gao X, Jiang W, Jiménez-Osés G, Choi MS, Houk KN, Tang Y, Walsh CT. An iterative, bimodular nonribosomal peptide synthetase that converts anthranilate and tryptophan into tetracyclic asperlicins. Chem Biol 2013;20:870–878.

30. Gao X, Haynes SW, Ames BD, Wang P, Vien LP, Walsh CT, Tang Y. Cyclization of fungal nonribosomal peptides by a terminal condensation-like domain. Nat Chem Biol 2012;8:823–830.

31. Hühner E, Backhaus K, Kraut R, Li S-M. Production of α-keto carboxylic acid dimers in yeast by overexpression of NRPS-like genes from *Aspergillus terreus*. Appl Microbiol Biotechnol 2018;102:1663–1672.

32. Tsunematsu Y, Ishikawa N, Wakana D, Goda Y, Noguchi H, Moriya H, Hotta K, Watanabe K. Distinct mechanisms for spiro-carbon formation reveal biosynthetic pathway crosstalk. Nat Chem Biol 2013;9:818–825.

33. Yu D, Xu F, Zi J, Wang S, Gage D, Zeng J, Zhan J. Engineered production of fungal anticancer cyclooligomer depsipeptides in *Saccharomyces cerevisiae*. Metab Eng 2013;18:60–68.

34. Siewers V, San-Bento R, Nielsen J. Implementation of communication-mediating domains for non-ribosomal peptide production in *Saccharomyces cerevisiae*. Biotechnol Bioeng 2010;106:841–844.

35. Wehrs M, Prahl J-P, Moon J, Li Y, Tanjore D, Keasling JD, Pray T, Mukhopadhyay A. Production efficiency of the bacterial non-ribosomal peptide indigoidine relies on the respiratory metabolic state in *S. cerevisiae*. Microb Cell Fact 2019;18:218.

36. Takahashi H, Kumagai T, Kitani K, Mori M, Matoba Y, Sugiyama M. Cloning and characterization of a *Streptomyces* single module type non-ribosomal peptide synthetase catalyzing a blue pigment synthesis. J Biol Chem 2007;282:9073–9081.

37. Xu W, Cai X, Jung ME, Tang Y. Analysis of intact and dissected fungal polyketide synthase-nonribosomal peptide synthetase in vitro and in *Saccharomyces cerevisiae*. J Am Chem Soc 2010;132:13604–13607.
